# Cytomegalovirus Replicon-Based Regulation of Gene Expression *In Vitro* and *In Vivo*

**DOI:** 10.1371/journal.ppat.1002728

**Published:** 2012-06-07

**Authors:** Hermine Mohr, Christian A. Mohr, Marlon R. Schneider, Laura Scrivano, Barbara Adler, Simone Kraner-Schreiber, Angelika Schnieke, Maik Dahlhoff, Eckhard Wolf, Ulrich H. Koszinowski, Zsolt Ruzsics

**Affiliations:** 1 Max von Pettenkofer-Institute, Ludwig-Maximilians-Universität München, Munich, Germany; 2 Institute of Molecular Animal Breeding and Biotechnology, Ludwig-Maximilians-Universität München, Munich, Germany; 3 Chair of Livestock Biotechnology, Technische Universität München, Freising, Germany; University of Southern California Keck School of Medicine, United States of America

## Abstract

There is increasing evidence for a connection between DNA replication and the expression of adjacent genes. Therefore, this study addressed the question of whether a herpesvirus origin of replication can be used to activate or increase the expression of adjacent genes. Cell lines carrying an episomal vector, in which reporter genes are linked to the murine cytomegalovirus (MCMV) origin of lytic replication (oriLyt), were constructed. Reporter gene expression was silenced by a histone-deacetylase-dependent mechanism, but was resolved upon lytic infection with MCMV. Replication of the episome was observed subsequent to infection, leading to the induction of gene expression by more than 1000-fold. oriLyt-based regulation thus provided a unique opportunity for virus-induced conditional gene expression without the need for an additional induction mechanism. This principle was exploited to show effective late trans-complementation of the toxic viral protein M50 and the glycoprotein gO of MCMV. Moreover, the application of this principle for intracellular immunization against herpesvirus infection was demonstrated. The results of the present study show that viral infection specifically activated the expression of a dominant-negative transgene, which inhibited viral growth. This conditional system was operative in explant cultures of transgenic mice, but not *in vivo*. Several applications are discussed.

## Author Summary

All herpesviruses show a precisely regulated gene expression profile, including true-late genes, which are turned on only after the onset of DNA replication. We used this intrinsic viral mechanism to generate a versatile conditional gene expression system that exploits the activity of the murine cytomegalovirus (MCMV) viral origin of lytic replication (oriLyt). Upon virus infection, replication of the viral genome also led to the replication and activation of the oriLyt-coupled episomal transgene. The oriLyt-based replicons were silenced in all stable cell lines and transgenic mice; however, virus infection liberated the plasmids from histone-deacetylase-induced inactivation. As maximum gene expression relied on relief from silencing via replication of the episomal constructs, very strong induction of the reporter gene was achieved. We showed that this system can be used for trans-complementation of late, toxic viral genes, to block virus production by activating dominant-negative (DN) transgenes, and to provide a new tool to study the principles of viral replication.

## Introduction

Herpesviruses are among the most complex DNA viruses, encoding up to 200 genes. The characteristic temporal control of viral gene expression ensures the appropriate regulation of the morphogenic process. Transcription factors, activated immediately upon virion entry into cells or after reactivation of latent viruses, regulate the expression of early genes and start a cascade of transcription of early-late, leaky-late and true-late genes [1]. Remarkably, in contrast to leaky-late genes, expression of true-late genes begins only after viral DNA replication, and can be blocked by inhibitors of the viral DNA polymerase such as phosphonoacetic acid (PAA) [2]. As the expression of true-late genes depends upon viral replication, the promoters of late genes were analyzed in the context of origin of lytic replication (oriLyt) sequences. Notably, isolated true-late promoters encoded by plasmids or integrated into the cellular genome respond like early promoters upon infection. Yet, the same promoters, together with an oriLyt provided in *cis*, restore the typical late expression patterns. This property was first demonstrated for HSV-1 [3], but was confirmed for several other herpesviruses [4], [5]. Six conserved replication proteins comprise the core components of lytic DNA replication and are shared by all herpesviruses [6]. To a certain degree, the core replication machinery can be complemented by another herpesvirus [7]. Yet, closely-related cytomegaloviruses (CMV) oriLyts are not exchangeable; the human CMV oriLyt cannot be activated by murine CMV or simian CMV [8], [9].

Two models have been proposed to explain the mechanism of lytic DNA replication. One includes circularization of the parental linear viral genome in the nucleus followed by an initial bidirectional theta-replication followed by a rolling circle mechanism [10]. An alternative model suggests a biphasic process in which DNA replication is initially induced by an origin-binding protein, which then recruits the replication machinery to the oriLyt sequences [11]. Later, it switches to a recombination-dependent replication and/or rolling circle mechanism [12], in which D-loop formation contributes to replication initiation [13]. This model is reflected by the highly branched concatemeric genomes observed in herpesvirus DNA replication.

Lytic replication origins have been identified by transient plasmid replication assays [6]. In these studies, cells transfected with plasmids containing putative oriLyt sequences were infected with the respective viruses. Under these transient conditions, plasmids containing an oriLyt were replicated by the viral machinery provided *in trans*. Stable maintenance of oriLyt-containing plasmids has been described for herpesvirus amplicon vectors. Herpes viral amplicon vectors contain an oriLyt, a gene (or genes) and, importantly, packaging signals for vector DNA incorporation into herpesvirus virions [14]. Standard amplicon vectors are diluted in dividing cells, yet hybrid vectors containing additional elements such as EBNA1/oriP or surface/matrix attachment region (S/MAR) sequences enhance stability. While expression of transgenes delivered to target cells has been analyzed in several studies, the potential impact of the presence of the oriLyt on transgene expression has not been addressed.

Conditional gene expression systems are important tools in virology and help to avoid toxicity and related problems. Inducible systems are usually based on additional elements that provide regulatory factors such as the Tet-system, or on recombination-dependent gene expression, such as the Cre/loxP mediated system. It is important to note that many viral promoters can also be trans-activated upon virus infection; however, their induction usually follows early or early-late kinetics and ranges only in the linear scale [15] and, thus, compares poorly with the viral expression levels and kinetics shown by late genes. Therefore, the aim of the present study was to construct an inducible expression system, which is stably maintained in the host cell and allows strong induction of late genes.

Although replication of a DNA fragment containing a lytic origin of HSV-1 (oriS) was initiated by superinfection [16], other studies show contradictory results [17], suggesting effects due to the host chromatin environment. To avoid this, we used the pEPI-1 vector as a backbone. The pEPI vectors are non-viral episomes that are maintained by an S/MAR element and replicate semi-conservatively once per cell cycle. These vectors can be maintained in many cell types without selection [18], [19]. Also, maintenance of the episome in mouse cell lines did not prevent silencing of the encoded transgene [20].

Here, we show that transcription units that are combined with an MCMV-oriLyt encoded on the pEPI background are indeed silenced. Yet, specific induction of DNA replication and gene expression both occurred after viral infection. Most notably, this system proved to be very useful for protein trans-complementation of defective viruses and studies of dominant-negative (DN) viral function.

## Results

### Mechanism of replication-dependent gene induction

A recurrent problem in transgenesis is the phenomenon of gene silencing [21]. Yet, gene silencing might be advantageous if constitutive gene expression is not favored. To obtain a gene expression system that is induced by infection by specific viruses, we relied on the fact that herpesviruses activate gene expression in *trans*, interfere with the host silencing machinery [22], [23], and will replicate constructs containing an oriLyt [9], [24]. Therefore, we established a novel CMV-inducible gene expression system in which natural silencing of the gene of interest in the transgenic cell is obtained. DNA replication after CMV infection then liberates the transgene, leading to the powerful induction of expression (Supp. **Fig. S1C**).

### Construction of the replicon vector pEpibo-luc-ori

MCMV was chosen because it is well characterized and facilitates *in vivo* investigation. Furthermore, the position of the minimal oriLyt of MCMV has already been defined [9]. It is located within a complex and highly structured locus containing several palindromes, inverted and direct repeats, and transcription factor binding sites. It has been shown for other herpesviruses that additional sequences flanking the minimal oriLyt can enhance replication; therefore, we decided to clone a 3.9-kb fragment containing the minimal 1.7-kb oriLyt fragment and adjacent sequences. Due to its size and the presence of various repeats, PCR amplification of the template was not feasible. So, we cloned the oriLyt fragment using a new BAC-based pick-up strategy (Supp. **Fig. S1A**) and inserted it into the episomal vector, pEpibo. To test the replicon vector, we used firefly luciferase (FL) as a reporter and measured the induction of gene expression in response to oriLyt activation by creating the vector, pEpibo-luc-ori (Supp. **Fig. S1B**). Here, FL expression was driven by the minimal SV40 promoter, which is not induced upon infection with MCMV (Supp. **Fig. S2**).

### Reporter gene expression from the oriLyt-replicon is inactivated by histone-deacetylase-dependent silencing and is resolved by MCMV infection

**Figure 1:**
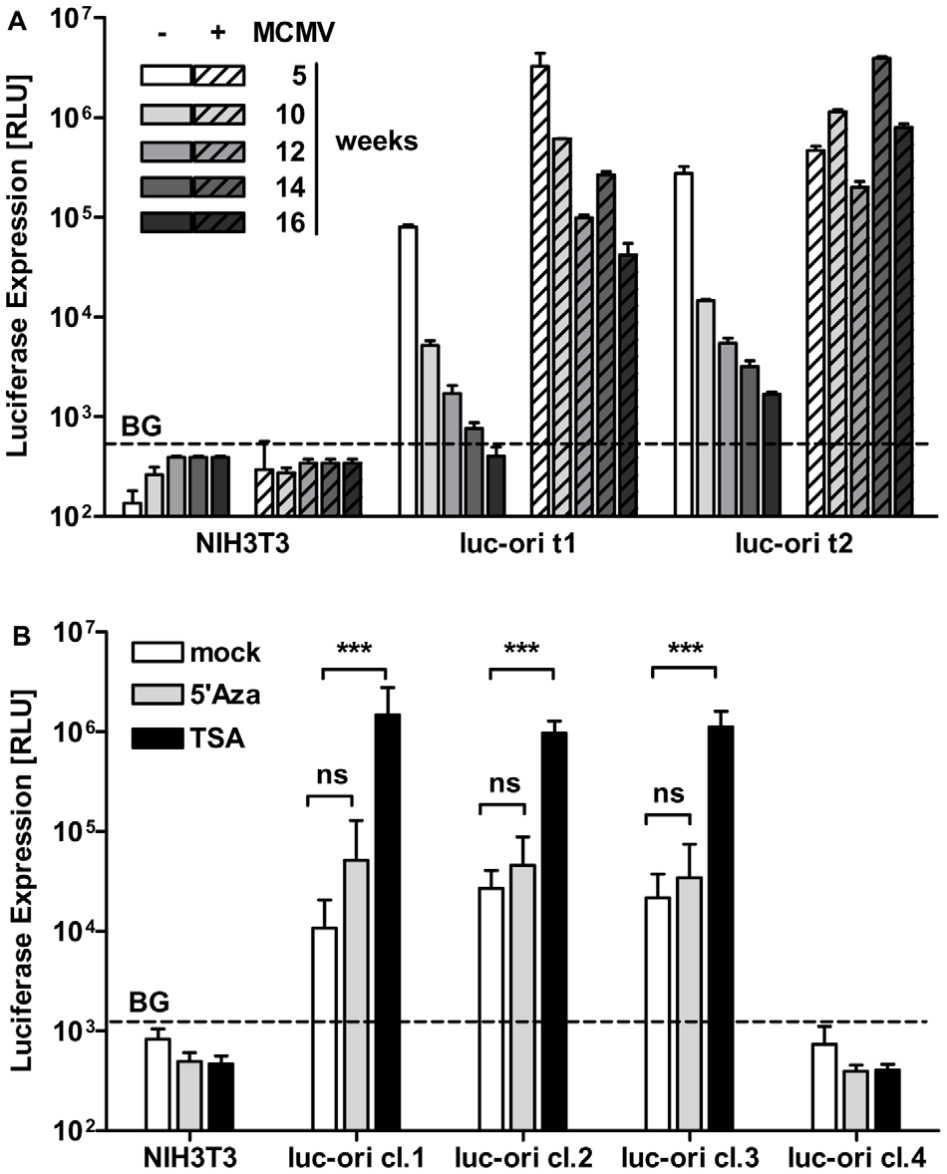
Infection with MCMV reactivates silenced replicon vector encoded reporter gene expression. (A) In two stable NIH3T3 cell pools transfected with pEpibo-luc-ori (luc-ori t1, luc-ori t2) expression of FL was measured in absence of infection (plain bars, - MCMV) at the indicated weeks after transfection. Reporter gene expression is lost in uninfected cells over time. At the same time points the pools were infected with MCMV at an MOI of 0.5 (hatched bars, +MCMV). 24 h p.i. of both luc-ori t1 and t2 with high expression of FL was induced. NIH3T3 fibroblasts served as a control to determine background signal (BG) of the luciferin substrate. (B) Vector pEpibo-luc-ori was inactivated by histone deacetylation. Four cell clones (cl. 1–4) derived from subcloning of luc-ori t1 were subjected to treatment with 25 µM 5′ aza-cytidine (5′Aza, gray bars), an inhibitor of CpG-methylation, 330 nM Trichostatin A (TSA, black bars) for 36 h or left untreated (mock, open bars). FL expression was analyzed in comparison to parental NIH3T3 cells. FL expression was significantly enhanced by de-condensing histone packaging through TSA treatment (*** p<0.001, ns p>0.05, Two-Way-Anova, depicted is mean+SD). RLU (relative light units), p.i. post infection, weeks = weeks post transfection of pEpibo-luc-ori.

To characterize the gene expression driven by pEpibo-luc-ori, two independent pools of NIH3T3 transfectants were generated. In the absence of infection, FL expression decreased to the limit of detection after 16 weeks in both pools (luc-ori-t1 and -t2). However, infection with MCMV restored FL expression by 3 to 4 orders of magnitude in both transgenic cell pools, irrespectively of the treatment time (**Fig. 1A**). Thus, loss of FL expression due to silencing was recovered upon virus infection.

There are several different mechanisms of transgene silencing [21]. We wanted to determine whether the construct was inactivated by *de novo* methylation of the promoter sequences, or whether chromatin condensation was the main contributor. We added specific inhibitors to cell clones derived from the cell pool, luc-ori t1, and measured FL expression after 36 h. Three sub-clones (cl.1, cl.2, cl.3) showed a basal level of FL expression and one clone was negative (cl.4) before treatment. Inhibition of CpG-methylation by 25 µM 5′-aza-cytidine [21] did not enhance expression, but treatment with 330 nM trichostatin A (TSA) resulted in about 100-fold induction in cl.1, cl.2 and cl.3 (**Fig. 1B**) . Efficiency of 5′ aza-cytidine treatment was controlled by recovery of methylation-dependent de-silencing of GFP fluorescence (Supp. **Fig S3A**). Recovery of FL induction by TSA was dose-dependent, but achieved induction levels lower than those obtained by infection with MCMV (Supp. **Fig S3B**). Furthermore, we found only limited co-operativity of TSA and MCMV infection on the induction of FL expression (Supp. Fig S4B). Western Blot analysis of MCMV infected cells treated with TSA revealed an enhancement of immediate early gene expression as previously described [25], but it disturbed gene expression at late time points (Supp. Fig S4A). Accordingly, 330 nM TSA inhibited MCMV production by 100-fold in a multi-step growth curve analysis (Supp. Fig S4B). Thus, MCMV infection could enhance FL signals induced by TSA, but was inhibited itself with increasing TSA concentrations. Consequently, the highest FL induction was achieved in infected cells without TSA treatment (Supp. Fig S4C). Interestingly, FL expression by clone cl.4 was not detectable under any conditions; this may have been due to the lack of an intact pEpibo-luc-ori episome or integration. As TSA is a potent inhibitor of histone deacetylases (HDACs) [26], these results strongly suggest that pEpibo-luc-ori driven gene expression is constrained by histone modifications other than DNA methylation.

### Activation of the replicon is specific to infection with the respective virus

**Figure 2:**
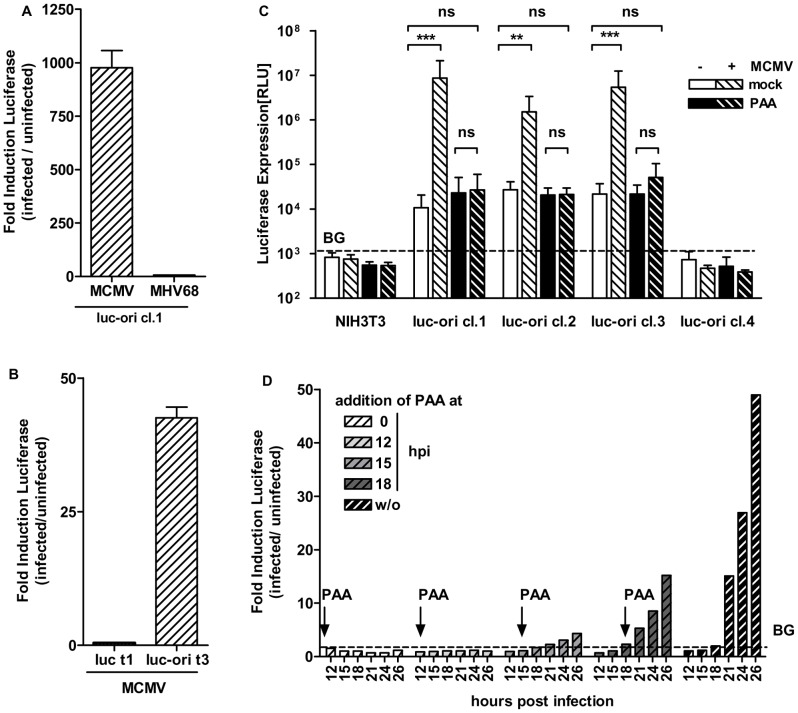
Induction of replicon vector is dependent on the MCMV DNA replication. (A) Induction of MCMV oriLyt is specific to MCMV infection. Cell line luc-ori cl.1 was infected with MCMV (beta herpesvirus; MOI = 0.5) or murine herpesvirus 68 (MHV68, gamma herpesvirus; MOI = 0.5) or left untreated. 36 h p.i. a bioluminescence assay was performed and the induction of the FL was calculated as the ratio of RLU of infected to uninfected cells. (B) NIH3T3 cells were stably transfected with pEpibo-luc (luc t1) or pEpibo-luc-ori (luc-ori t3). Depicted is the ratio of FL expression of the resulting cell pools before and after infection with MCMV at an MOI of 1. In the luc t1 cell pool, lacking the oriLyt sequence, FL expression is not enhanced by infection, in contrast to the luc-ori t3 cell pool, in which FL expression is induced about 40 fold at 36 h p.i. (C) NIH3T3 or luc-ori cell lines (luc-ori cl.1–cl.4) were infected with MCMV at an MOI of 0.5 (hatched bars) or left uninfected (plain bars). In addition, cell lines were either treated with phosponoacidic acid (PAA, 300 µg/ml, black bars) or left untreated (white bars). 36 h p.i. FL induction was measured via bioluminescence assay. While reporter expression is induced up to 1000-fold in MCMV infected cells lines cl.1–3, inhibition of viral DNA polymerase by PAA blocks the induction of FL expression. (D) Induction can be inhibited with PAA completely if it is added before replication has started and can be reduced if added after the onset of DNA replication. Cell line luc-ori cl.1 was infected with MCMV at an MOI = 0.5 or left uninfected. Arrows indicate time points when 300 µg/ml PAA was added to the cells. Cells were harvested at indicated time points and FL was measured via bioluminescence assay. Induction of the FL was calculated as the ratio of RLU of infected to uninfected cells. (p.i., post infection; BG = Background; ***: p<0.001, ns: p>0.05, Two-Way-ANOVA, depicted is mean+SD).

We then asked whether virus specificity, namely infection with MCMV, is necessary to activate FL from the replicon vector. As the core replication machinery is conserved in herpesviruses, we determined whether a herpesvirus from another subfamily could induce gene expression by the MCMV oriLyt system. As NIH3T3 cells are also permissive for the MHV68 γ-herpesvirus, which induces lytic replication in almost all infected mouse cells, we examined infection of an isolated cell clone, luc-ori cl.1, with MHV68 and MCMV (**Fig. 2A**). Whereas infection with MCMV resulted in typically high induction of FL expression (by three orders of magnitude), infection with MHV68 resulted only in a marginal increase of (∼5–10-fold). Because only MCMV was able to fully activate expression of the MCMV-oriLyt-replicon, the induction of the replicon system appears to be specific for infection with the corresponding herpesvirus.

### Induction of luciferase expression and de-silencing of pEpibo-luc-ori requires viral DNA replication

In view of the fact that silencing of pEpibo-luc-ori was lifted upon MCMV infection, we analyzed the role of the oriLyt element. To this end we constructed pEpibo-luc, which lacks the oriLyt sequence but is otherwise identical to pEpibo-luc-ori. NIH3T3 cells transfected with the pEpibo-luc generated cell pool, luc t1, and NIH3t3 cells transfected with pEpibo-luc-ori generated cell pool, luc-ori t3 (**Fig. 2B**). Silencing of constitutive FL expression was observed in both cell lines over time (data not shown). Yet, infection with MCMV enhanced the reporter signal selectively in the oriLyt-containing cell line, luc-ori t3, but not in the oriLyt negative cell line, luc-t1. It is important to note that oriLyt is located downstream of the multiple cloning sites into which the transgene/FL was inserted; thus, transgene transcription cannot be activated by means of cryptic promoter regions. This confirms the key role played by the oriLyt element in MCMV infection-dependent activation of FL expression in the luc-ori cell lines.

Next, we examined whether induction of the *luc* gene in pEpibo-luc-ori requires DNA replication of the infecting MCMV. Cell clones, luc-ori cl.1–cl.4 were infected with MCMV in the presence or absence of PAA, a specific inhibitor of the viral polymerase [27]. In the absence of PAA, FL expression in cell clones luc-ori cl.1–cl.3 increased by 100–1000-fold upon infection with MCMV (**Fig. 2C**). However, in presence of PAA, FL expression was similar to that in uninfected cells. PAA alone, in absence of infection, had no influence on FL expression. These data clearly show that induction of pEpibo-luc-ori expression upon MCMV infection was inhibited by PAA. Thus, MCMV-induced de-silencing and induction of FL was dependent on viral DNA replication. Cell line luc-ori cl. 4 did not respond to any treatment as already seen in the TSA experiments. Induction of FL in the controls began at the onset of DNA replication and increased exponentially over time (**Fig. 2D**; dark hatched columns). Notably, PAA was only able to inhibit induction of the oriLyt system when added prior to the onset of DNA replication, which starts around 8–12 h p.i. in MCMV [28], [29](**Fig. 2D**). If PAA was added after the initiation of replication, its effect on FL expression levels waned, further supporting the contribution of viral DNA replication. FL induction after infection proved to be a very robust property of the cell lines, as the level of induction was maintained in luc-ori cl.1 cells even after more than 6 months of continuous culture (data not shown).

**Figure 3:**
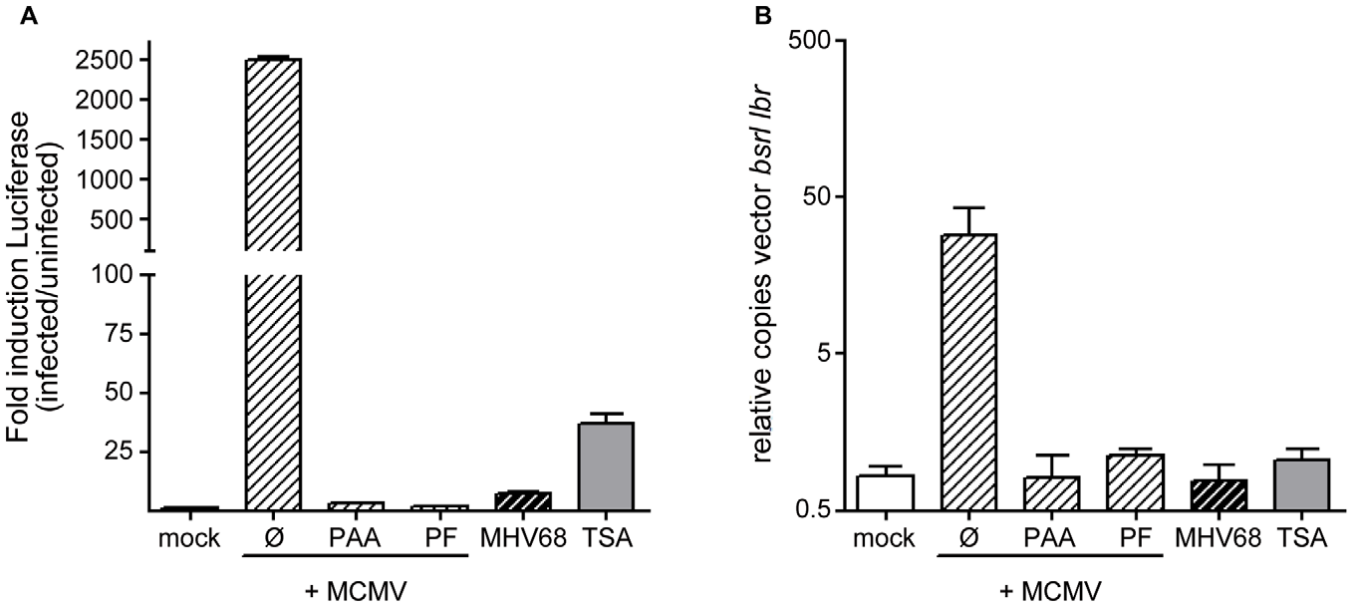
pEpibo-luc-ori is amplified upon MCMV infection. luc-ori cl.1 cells were infected with MCMV (white hatched bars), MHV68 (black hatched bars) at an MOI of 1 or left untreated (white bar, mock), or treated with 330 nM TSA. In addition, the DNA replication inhibitors PAA (300 µg/ml) and PF (100 µg/ml) were added to infected cells. (A) Bioluminescence assay was performed to determine the FL induction and (B) quantitative realtime PCR was performed 36 h p.i. to determine copy numbers of pEpibo-luc-ori vectors by a PCR specific for the *bsr* coding sequence compared to the cellular gene lamin B receptor (*lbr*).

Because induction of reporter gene expression from the vector was dependent on the oriLyt sequence (**Fig. 2B**), quantitative PCR (qPCR) experiments were performed to determine whether the episomal vector was amplified during infection. We measured the relative pEpibo-luc-ori copy numbers in infected and uninfected cells normalized to the endogenous murine *lbr* gene. Induction of FL expression (**Fig. 3A**) correlated with a significant increase in the pEpibo-luc-ori copy number (about 50-fold; **Fig. 3B**). Notably, in the cell line, luc-ori cl.4, in which MCMV infection did not de-silence FL expression, there was no increase in the copy number of the replicon vector sequence (Supp **Fig. S5)**. Phosphonoformic acid (PF), like PAA, is a specific inhibitor of herpesvirus DNA polymerases. As expected, no amplification of the vector was detected after PAA or PF treatment of infected luc-ori cl.1 cells (**Fig. 3B**), whereas infection of luc-ori cl.1 cells in the absence of inhibitors resulted in a ∼2500-fold increase in FL induction and a ∼50-fold increase in replicon vector amplification. By contrast, liberation of the vector from silencing by treatment with 300 nM TSA, without additional vector replication, resulted in only a moderate ∼25–30 fold induction of FL in luc-ori cl.1 cells (**Fig. 3A, B**).

### OriLyt-inducible activation of gene expression trans-complements late viral proteins

Trans-complementation of late gene defects by cell lines constitutively expressing the gene of interest is sometimes challenging. Incorrect timing, insufficient expression levels, aberrant intracellular distribution of isolated proteins, or toxicity of the constitutively expressed viral transgenes, can complicate cell based trans-complementation systems [30]–[32]. In particular, high constitutive expression of viral glycoproteins is often toxic to cells [33], [34]. As the replicon vector was maintained with low basal, or even undetectable, levels of transgene expression, we hypothesized that this system may be suitable for trans-complementation of late gene defects. To examine this, we constructed two cell lines, one encoding a glycoprotein (gO) and the other encoding the protein M50, which is known to be toxic after isolated expression [30]. This protein performs an essential function during export of the nascent viral capsid from the nucleus to the cytoplasm.

**Figure 4:**
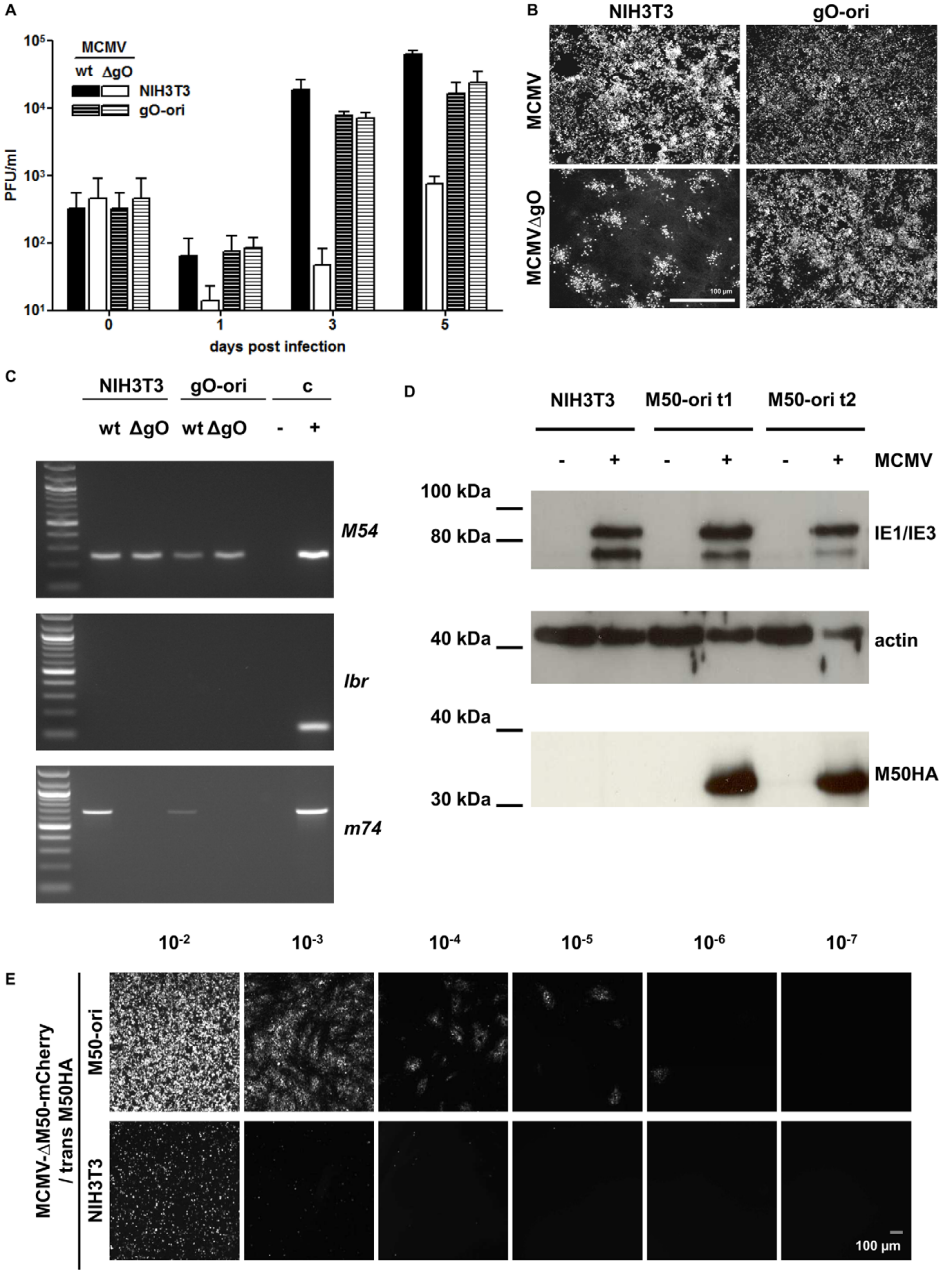
Replicon vector based trans-complementation. A–C trans-complementation of a glycoprotein. (A) Trans-complementation of the late glycoprotein gO can be facilitated by oriLyt-induced gene expression. NIH3T3 (striped bars) or gO-ori (plain bars) cell lines have been infected with MCMV-wt (white bars) or MCMVΔgO (black bars) at an MOI = 0.05 and centrifugal enhancement. At the indicated time points the number of the infectious virus was quantified in the culture supernatants by standard plaque assay. (B) Immunofluorescence microscopy of infected NIH3T3 or gO-ori cells performed 5 days post infection (MOI = 0.05). Infected cells were stained with the anti-IE antibody CHROMA-101. While MCMVΔgO is restricted to focal cell-to-cell spread in NIH3T3 fibroblasts, it spreads like wild type in the trans-complementing cell line gO-ori. (C) PCR analysis of virus progeny produced on gO-ori cell line. Viral DNA was isolated from supernatants of the viral growth curve of (A) from day 5, cleared from residual cells, and analyzed by PCR. The virus polymerase gene, M54 served as a positive control for viral infection. The cellular gene lbr, served as a control for the lack of residual genomic DNA. PCR on the m74 gene, encoding gO, showed the presence of the gene in MCMV-wt and the lack of it in MCMVΔgO. No uptake and recombination of gO after passage over gO-ori cells could be detected. D–E Trans-complementation of M50, a protein essential for nuclear export of viral capsids. (D) Detection of the M50HA protein (∼35 kDa) in cell lysates of NIH3T3, M50-ori t1 and M50-ori t2. The respective cell lines were infected with MCMV-wt at an MOI of 1 and cell lysates harvested 36 h p.i. (E) Growth of MCMVΔM50-cherry on complementing and non-complementing cell line. Supernatant of the reconstitution of MCMVΔM50-cherry on M50-ori cl.2.1 was serial diluted and used to infect NIH3T3 or M50-ori cells. The trans-complemented virus MCMVΔM50-cherry/M50HA could spread in M50-ori cells, but produced only primary infection in NIH3T3.

We cloned the m74 gene, coding gO [35], into the oriLyt vector to generate the responding cell line NIH3T3:gO-ori (gO-ori). MCMV, like HCMV, lacking gO is restricted to focal spreading and the release of infectious virions into the supernatant is hampered [35]. In the cell line containing the gO-ori vector, however, infection with MCMV-ΔgO should lead to pEpibo-gO-ori replication and m74 gene expression; thereby reconstituting release of infectious progeny. MCMV-ΔgO released from NIH3T3 about ∼2–2.5 orders of magnitude less virus than MCMV-wt. By contrast, growth in the complementing cell line resulted in comparable titers for both MCMV-ΔgO and MCMV-wt (**Fig. 4A**). Thus, the virus defect was rescued and the virus was no longer restricted to a focal growth pattern (**Fig. 4B**). This confirms that the replicon expression system can be used to efficiently produce late viral proteins in *trans*. To determine whether the viral genome reverts to a wt-like virus due to recombination with the episomal replicon vector, PCR analysis of the m74 gene (gO) was performed using viruses harvested on day 5 from the supernatants used in the growth curve experiments. Supernatants were centrifuged to remove cells and the cellular DNA was digested with DNase to discriminate between the m74 gene in the replicon vectors in the cells and the viral genome. The fact that the supernatants were free from cellular debris was confirmed by the lack of the cellular gene *lbr*. Presence of viral genomic DNA was confirmed by amplifying the viral DNA polymerase, M54. Whereas all supernatants were positive for the M54 gene and negative for the *lbr* gene, m74 was detected only in MCMV-wt, and not in MCMVΔgO, regardless of whether it originated from NIH3T3 or gO-ori cells (**Fig. 4C**). Furthermore, we never observed any reversion of the phenotypic restriction of MCMVΔgO to cell-to-cell spreading (data not shown). No recombination was detected between the replicon vector and the MCMVΔgO virus.

To assess the suitability of the system for complementing a toxic protein, the M50 gene (including a C-terminal HA-tag) was cloned into the oriLyt vector to generate the cell line, NIH3T3:M50-ori (M50-ori). Previous attempts at generating trans-complementing M50 cell lines using conventional methods failed due to the toxicity of the M50 protein [30]. To assess M50HA expression in two M50-ori cell pools (M50-ori t1 and t2), Western blot analysis was performed using an HA- specific antibody. Protein loading was controlled by actin detection, and infection was controlled by detecting the viral immediate-early proteins, IE1/IE3. No M50HA protein was detectable in uninfected cells; however, infection of the cell pools resulted in de-silencing and strong expression of M50HA (**Fig. 4D**). To analyze trans-complementation and recombination, virus reconstitution from MCMV-BAC DNA lacking the essential M50 gene was performed. To this end, M50-ori t1 and M50-ori t2, as well as NIH3T3 cells and MEF cells, were transfected with the BAC pSM3fr-Δ1-16-ΔM50-F. This BAC harbors the deletion of most of the M50 ORF, however due to overlap with the M49 gene 75 aa of the C-terminus of M50 had to be left intact and is homologous to the sequence in the replicon vector. Plaque formation was detected 3 days post-transfection in both M50-ori pools (data not shown), but not in NIH3T3 cells or MEF cells. Full cell lysis occurred 5 days post-transfection in the M50-ori cell pools, whereas no viral progeny were detected in the cells lacking the vector. To assess the efficiency of the reconstitution, serially diluted supernatants were used to infect the cloned cell line M50-ori cl. 2.1, or NIH3T3 cells. Titration of the supernatant on M50-ori cl.2.1 cells harboring the M50-ori vector revealed high titers (∼2×10^7^ PFU/ml) of the trans-complemented MCMV-ΔM50-F/M50HA. The reconstituted virus caused plaque formation and virus spread on M50-ori cl. 2.1 cells in a concentration-dependent manner, whereas only the signal from first-infected cells was seen in non-complementing NIH3T3 cells, without spread or plaque formation (**Fig. 4E**). Thus, the replicon vector allowed efficient trans-complementation of the toxic essential late protein M50. This complementation occurred at the protein level since the virus was unable to spread in non-complementing cells. However, we found that infection with MCMVΔM50-F/M50HA at a very high MOI resulted in formation of a few plaques, even in MEF cells, indicative of some genetic reversion. In the M50-ori cell pools, we calculated the reversion rate to be about 1 out of 10^4^–10^5^ PFU (data not shown).

### A replicon encoded DN protein inhibits virus production *in trans*

**Figure 5:**
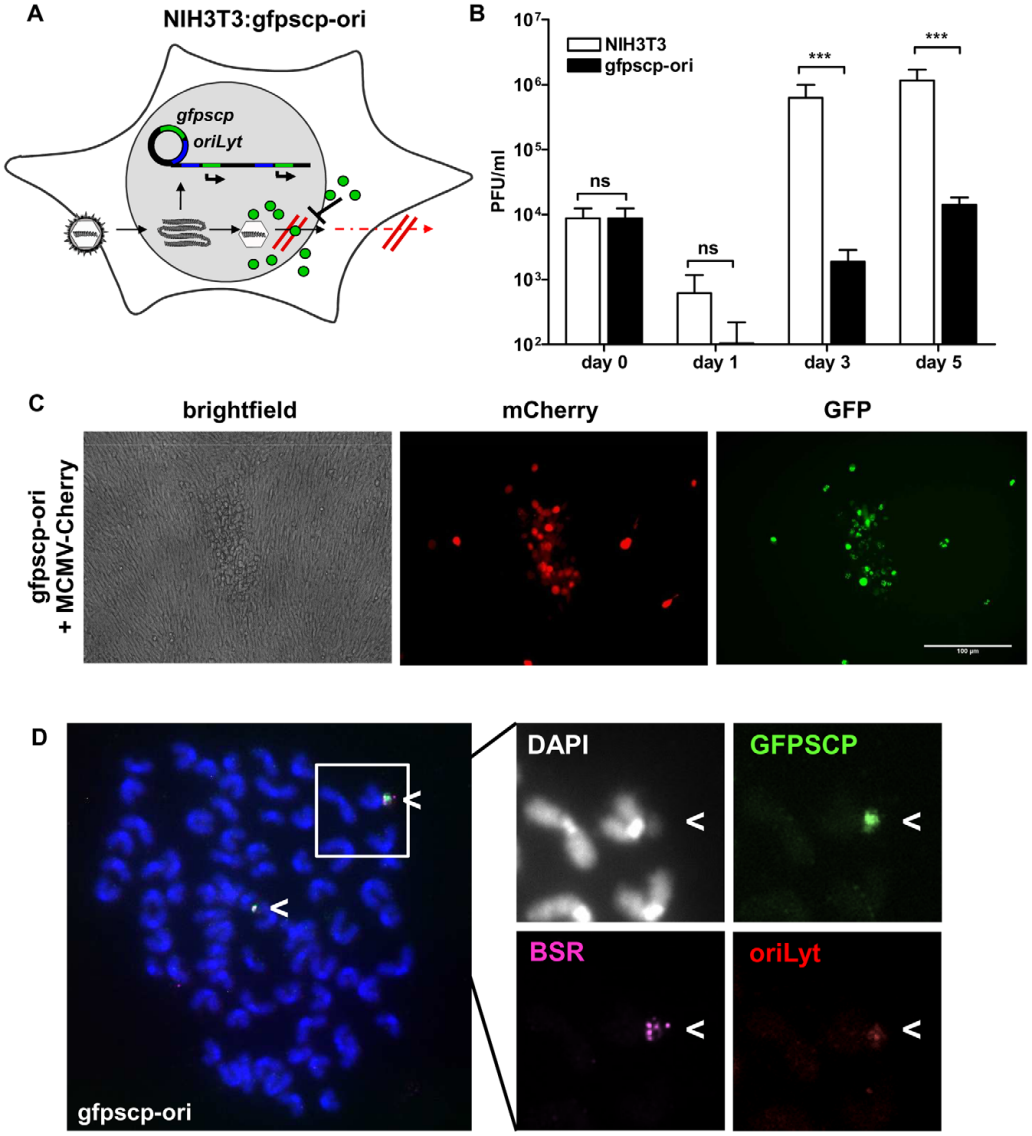
Induction of a dominant-negative protein by the replicon vector inhibits viral growth. (A) In the transgenic cell line NIH3T3: gfpscp-ori (gfpscp-ori), infection with MCMV induces replication of the construct and thereby activates the production of the dominant-negative protein GFPSCP (green symbols). This protein blocks egress from the nucleus and viral spread is inhibited. (B) NIH3T3 (white bars) and gfpscp-ori (black bars) cell lines were infected with MCMV-wt at MOI of 0.1. At the indicated time points, infectious virus was quantified in the supernatants by standard plaque assay. Due to the production of the inhibitory protein, the cell line gfpscp-ori releases 100–200 - fold fewer viruses into supernatants. (C) NIH3T3 or gfpscp-ori cell lines were infected with MCMV-mCherry at an MOI of 0.1 and expression of GFPSCP and mCherry was assessed 5 days p.i. by fluorescence microscopy. The mCherry protein is expressed with late kinetics and serves as an infection marker. Only in infected cells GFPSCP is produced and localizes according to its typical pattern in nuclear speckles. Plaques on the gfpscp-ori cell line are reduced in size. (D) FISH of metaphase spreads of uninfected gfpscp-ori cells (4n = 76). Three different probes complementary to the *gfpscp* gene (green), *bsr* gene (pink) and oriLyt (red) were used. Probes co-localized to DAPI stained extrachromosomal spots, indicating an episomal persistence of pEpibo-gfpscp-ori.

Next, we studied whether virus replication can be inhibited by a DN transgene induced late in the viral cascade. Inhibition of viral spread by the use of DN viral proteins, called intracellular immunization, was proposed by Baltimore in 1988 [36] and was inspired by the work of Friedman and colleagues [37]. They provided the proof-of-principle experiment showing that a truncated VP16 protein could reduce the replication of HSV-1 when stably expressed by the host cell line. Since then, toxicity caused by DN transgenes has been a problem [37]–[40]. To test the usefulness of our system for studying dominant-negative effects, we cloned the DN *gfpscp* gene [41], [42], which codes for a fusion between GFP and the small capsid protein (SCP) of MCMV, into the oriLyt vector. During infection of the NIH3T3:gfpscp-ori (gfpscp-ori) cell line, MCMV should de-silence expression of the inhibitory protein GFPSCP. Strong expression of GFPSCP should, in turn, block the egress of viral capsids from the nucleus (**Fig. 5A**).

To examine the inhibitory potential of the gfpscp-ori cell line, we performed growth curve analysis of MCMV in the inhibitory cell line and NIH3T3 cells. In contrast to DN expression *in cis* in the viral genome (which was about 1 million-fold reduced) [42], induced expression of the DN *in trans* resulted in inhibition of virus production by about 2 orders of magnitude only (**Fig. 5B**). This was also true for other cell lines such as M210-B4 (data not shown). However, complete inhibition could not be achieved, perhaps due to the fact that SCP represents the most abundant protein in viral capsids and, therefore, it is unlikely to be out-competed by the DN protein.

We then considered heterogenous de-silencing of the transgene in individual cells, either along with the cell cycle status or due to other reasons, which may result in cell subpopulations in which DN expression is absent. To address this possibility, we tested recombinant viruses derived from pSM3fr-Δ1-16-SCPiChe-FRT (MCMV-cherry). Here, the expression of mCherry served as a late infection marker as its expression starts concordant with that of the SCP gene. We observed a striking and specific correlation between infected cells and cells positive for GFPSCP fluorescence (**Fig. 5C**). Therefore, limited amounts of the DN protein rather than heterogeneous de-silencing and subsequent lack of the inhibitory protein in individual cells, is probably the cause of the limited DN effect.

To examine the transgene copy number prior to infection, fluorescence *in situ* hybridization (FISH) was performed. pEPI-1-based cell lines carry between 2–10 episomes per cell. FISH analysis of the *gfpscp*-ori cell line revealed approximately two episomal copies per cell (**Fig. 5D**) Integration events, as described for the original pEPI-EGFP [43] vector, were also detected. In 34 of the analyzed metaphase spreads, we identified 31 times episomal state of the pEpibo-gfpscp-ori vector. Additional integrated vectors were detected in five metaphase spreads. However, we did not find metaphase spreads associated with integrated vectors alone.

### A virus inducible oriLyt-dependent luciferase mouse (VIOLA)

**Figure 6:**
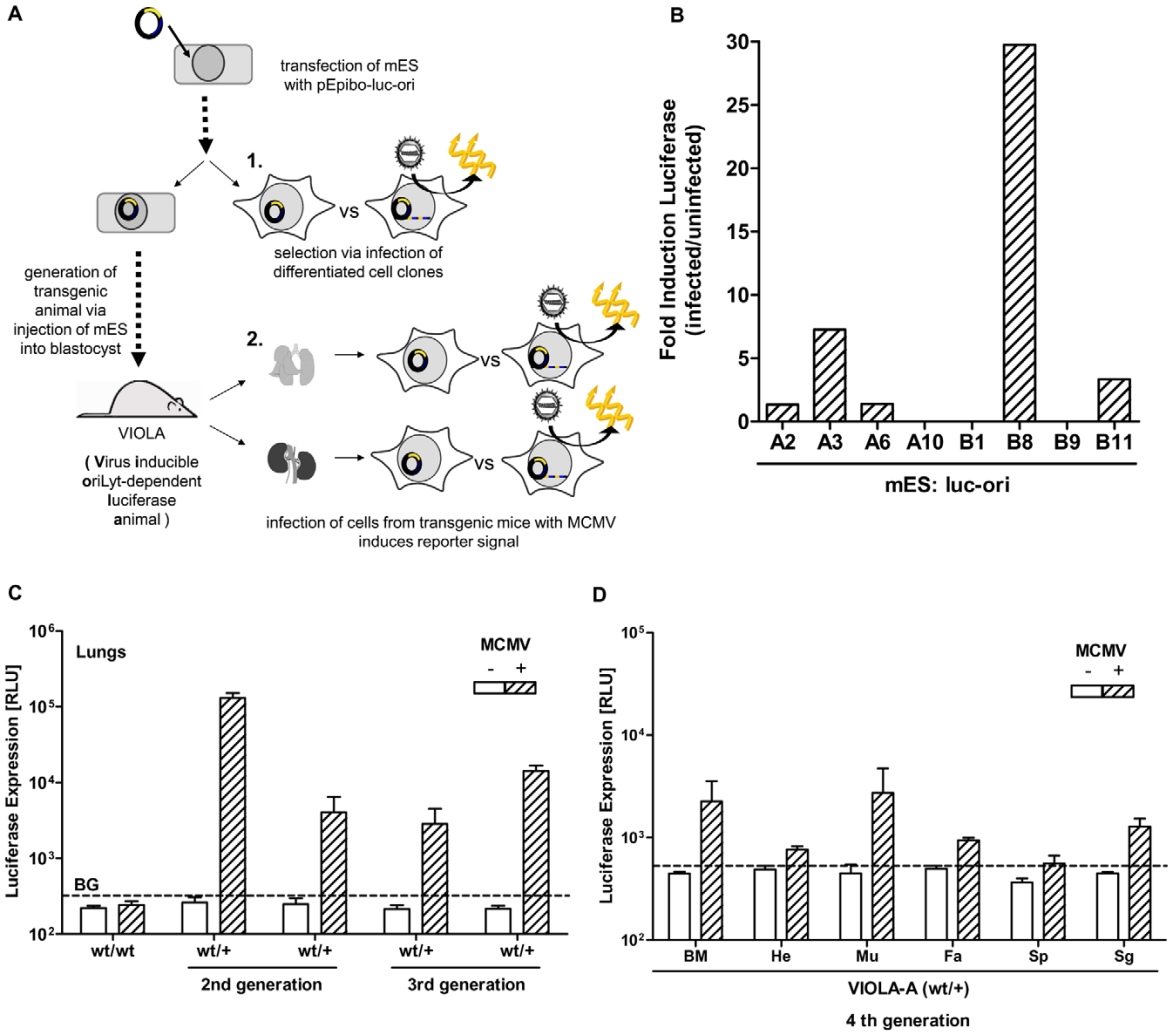
OriLyt dependent transgene expression in vivo. Generation of virus inducible oriLyt dependent luciferase animal (VIOLA). (A) (1.) mES cell clones were transfected with pEpibo-luc-ori and (2.) pre-selected for their induction capacity by MCMV infection *in vitro*. (3.) Mouse lines were analyzed for expression of FL before and after infection in explant cultures of different organs. (B) mES cells were transfected with pEpibo-luc-ori and 8 clones were isolated. A portion of the clones were partially differentiated for 3 weeks and then infected with MCMV at an MOI of 0.5 or left untreated. 36 h p.i. a bioluminescence assay was performed and the induction of the FL was calculated as the ratio of RLU of infected to uninfected cells. mES clones A3 and B8 were selected for generation of transgenic animals. (C) Explant cultures of lungs taken from mice of the Viola–A strain, backcrossed one (2^nd^ generation) or two (3^rd^ generation) times to 129X1/SvJ, were produced and were infected with MCMV-wt with an MOI of 0.5 (hatched bars) or left untreated (white bars). 24 h p.i. FL expression was determined by measuring relative light units (RLU). As a control mice of the background strain 129X1/SvJ (wt/wt) were used. (D) Explant cultures from bone marrow (BM), heart (He), muscle (Mu), fat (FA), spleen (Sp) and salivary glands (Sg) were grown from a VIOLA-A mouse of the fourth generation. Cells were infected with MCMV-wt with MOI of 0.5 (hatched bars) or left untreated (white bars). 24 h p.i. FL expression was determined by measuring relative light units (RLU). (p.i. = post infection ; BG = background of luciferin).

As already observed for several attenuated MCMV-mutants, the reduction in virus titer of 99% observed in tissue culture may result in even stronger attenuation if the system is operative *in vivo* [44]. To test the oriLyt system *in vivo*, we transferred the conditional DN principle to the natural host of MCMV, the mouse. To our knowledge, no transgenic mouse has been created based on the pEPI-1 vector. Therefore, to assess episomal stability and to test de-silencing *in vivo*, we generated transgenic mice carrying the pEpibo-luc-ori construct. This should permit the general concept to be studied *in vivo* (**Fig. 6A**). To this end, we transfected murine embryonic stem cells (mES line E14) with pEpibo-luc-ori and isolated eight cell clones. Note that mES cells are non-permissive for MCMV infection. To identify cell clones suitable for blastocyst injection, several cell populations were differentiated for 3 weeks to enable productive MCMV infection [45]. To study virus specific de-silencing in differentiated mES, the bioluminescence signal was measured both prior to and after MCMV infection. In three of the eight clones, no FL expression was detected under any conditions (A10, B1, B9); three other clones showed a weak increase in FL expression after infection (A2, A6, B11) and two clones were induced 7- (A3) and 30-fold (B8) (**Fig. 6B**).

The latter two clones (named line A and line B) were used for blastocyst injection. This led to at least one chimera for each line, which transmitted the luciferase gene to the next generation (lines were named VIOLA: virus inducible oriLyt-dependent luciferase animal). Basal FL expression and FL induction was assessed by measuring bioluminescence in lung explant cultures. Although the VIOLA-B line was positive for the *luc* gene, it did not express FL under any conditions (data not shown). Lung explants from 2^nd^ generation VIOLA-A animals expressed FL selectively after MCMV infection, and this property was maintained in subsequent generations (**Fig. 6C**). Induction of FL expression after MCMV infection was detected in each organ, whereas basal FL expression in uninfected cells was undetectable. FL induction was also observed in *ex vivo* explant cultures of other organs such as bone marrow, heart, muscle, fat, spleen, and salivary glands of 4^th^ generation animals (**Fig. 6D**). It is important to note that FL expression in these *ex vivo* cultures was merely a semi-quantitative estimate, as the explant cultures represent a heterogeneous pool of cells both permissive and non-permissive for MCMV infection. Therefore, although virus load was calculated as MOI = 0.5, viral infection could not be normalized.

To test whether virus-induced gene expression also operates *in vivo*, we infected VIOLA-A mice with 1×10^6^ PFU i.v. and measured the light signals using non-invasive imaging of whole animals over a period of 5 days. As a control, 129X1/SvJ mice were infected with 1×10^5^ PFU i.v. MCMV-luc [46]. Surprisingly, although the *luc* gene was activated in the explant cultures, no bioluminescence signal was detectable in the VIOLA mice *in vivo*. This should not be due to limited detection of the signal because the FL signal generated by the virus was easily detectable after infection with MCMV-luc at a 10-fold lower virus dose (Supp. **Fig S6A**). Furthermore, titration of the virus in organs of the infected VIOLA mice revealed a normal viral load. Therefore a failure to detect the FL signal in vivo is not due to reduced infectibility of VIOLA mice (Supp. Fig **S6B**). Southern blot hybridization of the transgenic mouse genomic DNA confirmed the presence of pEpibo-luc-ori; however, our data also showed that pEpibo-luc-ori sequences were integrated into the mouse genome (Supp. **Fig S7**). Integration of the replicon vector was also suggested by the fact that the inheritance pattern followed classical Mendelian rules. Thus, pEpibo-luc-ori was transferred to the transgenic mice, but the episomal state was not faithfully maintained.

## Discussion

In this study, a herpesvirus lytic origin of replication was combined with the transcription unit from an episomal vector to generate a novel inducible expression system, which is induced by infection with wt virus. In this system, herein referred to as the “replicon vector”, basal background gene expression was negligible as it was silenced by a histone deacetylase-dependent mechanism. However, upon viral infection, de-silencing and activation of the replicon vector led to a >1000-fold increase in induced gene expression. Neither genetic manipulation of the viral genome nor administration of any chemical compound was necessary. This replicon vector system may resolve difficulties encountered when trans-complementing late proteins (namely glycoproteins and toxic proteins), as shown by the trans-complementation of the viral glycoprotein O, and the toxic viral protein M50. Also, its utility for inhibiting virus multiplication was demonstrated, as shown by the use of DN proteins, such as GFPSCP.

Viral DNA replication was a prerequisite for strong reactivation of silenced transgenes as inhibition with PAA or PF abolished reporter gene expression. The presence of oriLyt in the replicon vector was, therefore, essential for induction of gene expression. Based on these data, we assume that oriLyt-mediated vector replication induced by MCMV is the key to gene expression. If there is further need for a true-late viral protein for activation (whose production would be also inhibited by PAA), it must also act in the context of the oriLyt sequence.

Induction of the MCMV replicon vector requires specific elements that are shared between the vector and the activating virus, since infection with MHV68 did not induce transgene expression. Although herpesviruses comprise a conserved core of replication proteins that can be exchanged between subfamilies [6], the activation or origin binding protein/s in β- and γ-herpesviruses is highly diverse between subfamilies and even within subfamilies. Although the mechanism of replication initiation by origin binding proteins has been extensively studied in α-herpesviruses and roseoloviruses, this knowledge is not yet available for other herpesvirus subfamilies. Interestingly, in CMV and γ-herpesviruses, proteins that work as transcriptional activators, such as UL84 for HCMV [47], Zta for EBV [48], [49] and K8 and Rta for KSHV [50], are also necessary for the initiation of viral DNA replication. In these cases, sequences have been identified that might act as promoters [51], , and a number of transcription factor binding sites were identified in several oriLyts [52]–[54]. In the present study, we can only exclude the effect of an intrinsic oriLyt promoter, as the reporter gene was placed upstream of the replication origin. However, the contribution of an enhancer function within the oriLyt sequence remains the subject of debate. Since induction was strongly dependent on DNA replication, enhancer activity alone cannot explain the results. Therefore, we assume that oriLyt-induced gene expression is due to: (a) conformational changes in DNA structure or histone packaging making the promoter for the reporter transgene more accessible to the transcription machinery, and/or (b) a marked increase in the number of templates available for transcription.

At first glance, the presence of both a viral oriLyt and transgenes appears similar to an amplicon vector construct, as the amplicon also contains both elements and, in addition, a pac signal. However, the oriLyt within amplicon vectors serves only to amplify the vector DNA for efficient packaging of a high number of vector copies into helper virus capsids via the pac signal [14]. Interestingly, the phenomenon of transgene silencing of amplicon vectors by HDAC-dependent mechanisms has also been described [55]. Whether or not the context of the oriLyt itself affects the level of gene expression has, to our knowledge, not been investigated. Neither has the question of oriLyt-specific de-silencing of the vector by super-infection of cells carrying the vector been addressed. Taking the results of our study into account, we expect that expression of transgenes provided by amplicons derived from human pathogens would be affected by natural super-infection. This prediction is testable using gene therapy studies incorporating herpesvirus amplicon vectors. The strength of this effect would probably depend on details such as transcription orientation and distance from the oriLyt sequence.

The replicon vector system for inducible gene expression should be applicable to all herpesvirus subfamilies. Moreover, most DNA viruses regulate late gene expression upon DNA replication, although it is achieved via different mechanisms. Such replicon vector systems may be applicable for polyomaviruses [56] and adenoviruses [57] with minor modifications. Notably, there are suggestions of a connection between the origins of replication and enhanced gene expression in higher eukaryotic genomes [58], [59].

Trans-complementation of late proteins involved in herpesvirus morphogenesis is still a difficult task. Incorrect timing, aberrant intracellular distribution due to missing viral interaction partners and incorrect expression levels of the viral protein may explain poor complementation results. For instance, the isolated expression of late herpesvirus promoters without providing an oriLyt in *cis* results in aberrant early expression of the transgene. Today, systems for inducible gene expression typically require the use of small chemical compounds such as tetracyclin or doxycyclin (as in the case of Tet-on/Tet-off systems) [60] or rapamycin (for FKBP12-based systems) [61]. In these systems, gene expression is activated synchronously and irrespective of the state of virus replication in all cells, whereas the oriLyt-based system uses viral DNA replication as the signal for induction. The expression of the gene increases in proportion with the amplification of the vector DNA and reflects the natural expression kinetics of late herpesvirus genes. As each cell is activated individually upon infection, the appropriate and correct timing of expression of the trans-complementing gene is determined by the infecting virus.

Trans-complementation carries the risk of recombination of genes provided *in trans* with the respective defective viral genome. We did not observe phenotypic or genotypic reversion of the ΔgO-phenotype. By contrast, we observed viable viruses at low levels in the M50-ori cell lines after reconstitution of a non-viable M50 deficient BAC. The high selection pressure for the production of infectious progeny favors the replication of M50 recombined viruses. In the replication competent gO-ori system, recombination only results in virus release; a phenotype with limited selective advantages. However, the rare reversion events observed during M50 complementation did not result in detectable reversion of the phenotype in first generation progeny (see Fig. 4E). Future studies are needed to identify the conditions that increase or decrease the risk of recombination using replicon vectors.

We also used the system for antiviral intracellular immunization. Constitutive expression of DN viral proteins in earlier studies [40] resulted in major defects in mice, such as substantial weight loss, probably due to cross-reaction between the dominant-negative viral transcription activators and cellular proteins, thereby altering natural gene expression profiles [40]. The expected advantage of replicon vectors lies in the fact that the DN protein is silenced. Thus, adverse effects caused by constitutive expression of toxic proteins are minimized. Our results show that we have been able to demonstrate proof-of-principle. It is not surprising that we could not achieve complete inhibition of viral replication in cell culture. A simple explanation is that the high abundance of wt SCP protein produced during infection (in terms of copy number per capsid) [62] simply out-competed the DN protein. One option may be to isolate clones containing high initial copy numbers of the replicon vector, or to replace the DN with that of a less abundant target protein such as M53 [63].

Episomal maintenance of pEPI has been shown in transgenic pigs, in which the pEPI-1 vector was not inactivated [64]. The present study reports, for the first time, the generation of transgenic mice using the pEPI replicon vector system. Functionality was testable in differentiated mES cell clones prior to generation of the transgenic lines. Starting from two responsive cell clones, two lines of VIOLA mice were generated. However, integration of the pEpibo-luc-ori vector was apparent in both VIOLA lines; although we cannot exclude the possibility that some episomal copies remained. Stabilization of the episomal properties could perhaps be improved by using murine S/MAR sequences instead of the human sequences used so far.

Remarkably, we found no evidence for gene induction *in vivo*, although reactivation of the reporter by MCMV was possible in *ex vivo* explant cultures. Controls indicated that the failure to detect the luciferase signal in living mice was not due to insufficient sensitivity. The failure of transgene induction *in vivo* perhaps reflects the chromatin conditions surrounding the integrated constructs. These conditions change under *in vivo* and *in vitro* conditions. Major epigenetic changes happen during *ex vivo* tissue culture [65], which might impinge on the strength of replicon vector silencing. Perhaps only metastable silencing conditions are lifted upon infection.

Virus induced replication and expression forms the basis of the here described replicon expression system. This represents a tool kit, which can be favorably utilized for studying herpesvirus DNA replication, trans-complementation, gene therapy vector production and genetic immunization *in vivo*.

## Material and Methods

### Ethics statement

All animal experiments were performed in strict accordance with German animal protection law (TierSchG) and approved by the responsible state office Regierung von Oberbayern (ROB) under protocol number 55.2-1-54-2531-195-09. The mice were housed and handled in an SPF condition in accordance with good animal practice and all efforts were made to minimize suffering as defined by Federation of European Laboratory Animal Science Associations (FELASA) and the national animal welfare body Gesellschaft für Versuchstierkunde - Society for Laboratory Animal Science (GV-SOLAS).

### Plasmids

The oriLyt of MCMV [9] was subcloned from an MCMV-bacterial artificial chromosome (BAC) using a pick-up-cloning strategy. A PCR fragment was amplified using primers H5′-MCMV-oriLyt-ori6kan-for (5′-GGCGGGAGCG ACGGGGGCGA GGCTGGAGAG ATCGTCGTCC GCCATGCTAG CACGCGTGCC AGTGTTACAA CCAATTAACC-3′) and H3′-MCMV-oriLyt-ori6kan-rev (5′-GAACGACCCC CGCTCCTGTA TAATTTCGAT GCCGGGGAGG TCGCCACGCG TCTGAAGATC AGCAGTTCAA CCTGTT-3′) containing homologies at H5′ to 91850–91894 bp and H3′ homologies at 91895–91939 bp of the MCMV-FRT BAC pSM3fr-FRT flanking a kanamycin resistance gene (kanR) and the bacterial oriR6K [66]. This PCR fragment was recombined by homologous recombination into pSM3fr-FRT as previously described [67]. Due to the insertion of a new *Nhe*I site (at the most upstream position in the inserted fragment) the oriLyt-containing genome segment could be excised together with the bacterial amplicon and the kanR marker by *Nhe*I digestion, as the next *Nhe*I site in the genome (at 95767 bp) was sited downstream of the oriLyt. The fragments generated by *NheI* digestion were recircularized by DNA ligation and used to transform PIR1 *E. coli* (Invitrogen). In PIR1 *E. coli*, only the oriR6K (and thereby the oriLyt-containing circularized *NheI* fragment) was maintained after kanamycin selection. The oriLyt fragment was subcloned from the resulting pO6kan-oriLyt plasmid by subsequent digestion with *Mlu*I and ligated into the *Mlu*I site of a modified pEPI-1 vector containing a blasticidin resistance gene (*bsr*) and an mOrange fluorescent marker (instead of the neomycin resistance marker and the *gfp* gene in the original vector) [68] to yield pEpibo-oriLyt.

An additional FL reporter gene, driven by the minimal SV40 promoter (48–250 bp) was excised from the pGL3-control plasmid (Promega, Acc# U47296) and inserted into the pEpibo-oriLyt vector upstream of the oriLyt via the *Kpn*I and *Xba*I restriction sites. The control plasmid, pEpibo-luc, was constructed by inserting the same fragment into the pEpibo vector (w/o oriLyt). To construct the pEpibo-GFPSCP-ori plasmid, the luciferase ORF was excised from pEpibo-luc-ori via the *HindIII* and *XbaI* sites and replaced with a PCR fragment containing the GFP-SCP ORF [41] flanked by the respective restriction sites. The vector pEpibo-gO-ori was generated by blunt-end cloning of the gO-ORF from pCR3-m74 [34] via Ecl126II into the *Hind*III and *Xba*I sites of pEpibo-luc-ori. To generate the pEpiNo-M50HA-ori vector, which contains a neomycin selection cassette instead of the blasticidin resistance gene, M50HA from pOriR6K-zeo-ie-M50HA [69] was cloned into the *Hind*III and *Xba*I sites of pEpiNo-luc-ori.

### Cells lines

Mouse embryonal fibroblasts (MEFs) and NIH3T3 cells (ATCC CRL-1658) were maintained in Dulbecco's modified Eagle's medium (DMEM) supplemented with 10% fetal calf serum (FCS), 100 units/ml penicillin, and 100 units/ml streptomycin. To generate stable cell lines, NIH3T3 cells were transfected in 6-well plates with 2.5 µg of the pEpibo construct using the TransIT-3T3 (Mirus) transfection reagent according to the manufacturer's protocol. Transfected cells were selected with 10 µg/ml blasticidin S (BS, Invivogen) or with 200 µg/ml G418 (Invivogen; for M50- ori) and were either studied as cell pools or as clones isolated by limiting dilution.

### Viruses, infections and growth analysis

The MCMV-wt and MHV68-wt viruses were derived from BACs pSM3fr and Rγ HV68A98.02, respectively [70], [71]. Reconstitution of the viruses, preparation of virus stocks and titrations were performed as described previously [72], [41]. To determine *in vitro* growth of MCMV in different cell lines, cells were plated at the same density and infected at an MOI of 0.1 (unless otherwise indicated). After 1 h of virus infection, cells were washed and fresh medium supplied. Supernatants of the infected cells were taken at the indicated time points (in triplicate) and titrated against MEFs using a standard plaque assay, or against the respective replicon cell line according to TCID_50_. Growth curve experiments were performed at least twice. MCMV-ΔgO virus production and trans-complementation were performed as described previously [34].

The M50-deficient virus was generated by modifying pSM3fr-Δ1-16-FRT [73]. First, to monitor late gene expression by MCMV, an expression marker was introduced into the 3′ UTR of the M48.2 gene, which encodes the small capsid protein (SCP). The internal ribosome entry site (IRES) of the encephalomyocarditis virus was derived from pIREShyg3 (Clontech, nt 1333–1924) and assembled upstream to the mCherry ORF from pmCherry-C1 (Clontech nt 597–1448). This IRES-mCherry cassette was then introduced into MCMV-BAC between nt 73570 and nt 73571 and directly after the M48.2 stop codon by recombineering [74] yielding pSM3fr-Δ1-16-FRT-SCPiChe. Next, the EGFP coding region from pEGFP-N1 (Clontech nt 679–1398) and the ampicillin resistance gene from LITMUS 28 (NEB nt 2764–1003) were assembled and introduced into pSM3frΔ1-16-FRT-SCPiChe between nt 75730–76451 via a second round of recombineering to yield pSM3fr-Δ1-16-ΔM50F. The newly-inserted cassette replaced most of the M50 ORF and allowed the EGFP ORF to be expressed instead of the deleted M50. The C-terminal 75 aa of M50, which overlap with the M49 gene, were left intact as described for the M50 deletion mutants [75].

### Generation of transgenic mice

To generate the VIOLA mouse line (virus inducible oriLyt-dependent luciferase animal), the murine embryonic stem cell line, mES E14, was transfected with pEpibo-luc-ori using an AMAXA nucleofector (Lonza Cologne, Germany) according to the manufacturer's instructions using program A-013. Transfected mES cells were cultured in mES cell medium (DMEM containing 15% FCS, 1% non-essential amino acids (Gibco) containing 1000 U/ml LIF (Millipore, Temecula, USA)) on mitomycin C-treated NIH3T3-bsr fibroblasts and selected with 5 µg/ml BS for 3 days. To select the appropriate clones, a proportion of the mES cells was differentiated by removing the LIF and feeder cells to allow productive infection with MCMV [44]. Appropriate clones were injected into C57BL/6 blastocysts, which were then implanted into a NMRI foster mother. The resulting chimeras were backcrossed to 129X1/SvJ mice (Jackson) to obtain the VIOLA line.

### Tissue explant cultures

For the explant cultures, the lungs, heart, muscle, fat and salivary glands were excised from individual mice, washed with PBS and minced in dissociation buffer (12.5 mM HEPES, 200 U/ml DNaseI and 13 Wünsch U/ml Liberase TM in PBS). Tissues were incubated at 37°C until a smooth homogenate was obtained. The homogenates, as well as the spleens and bone marrow cells (prepared from the femurs) were passed through a 100-µm strainer and resuspended in DMEM. Lung cells were resuspended in LSGS medium (DMEM containing, 15% FCS, 1% non-essential amino acids (NEAA), 1 µg/ml hydrocortisone, 10 ng/ml human epidermal growth factor, 3 ng/ml basic fibroblast growth factor, 10 µg/ml heparin). Heart, muscle, salivary gland and fat cells were cultured in DMEM containing 15% FCS, 1% NEAA and 50 µM 2-mercaptoethanol. Spleen cells were cultured in RPMI1640 containing 10% FCS, 1% L-glutamine and 10 mM HEPES. All cells other than muscle were plated on gelatin-coated culture flasks. Muscle cells were grown on collagen type V-coated culture flasks.

### Luciferase assays

To quantify FL expression, 100,000 cells/well were seeded into 12-well plates (in triplicate). After 4 h, the cells were either infected with MCMV or mock-treated. At 24 or 36 h after infection, the cells were carefully washed with PBS and lysed with 200 µl 1× Passive Lysis Buffer (Promega). The luciferase activity in the lysates (10 µl) was measured in a 96-well luminometer (Berthold). Experiments were performed at least three times. For the analysis of the silencing mechanism, cells were treated with 330 nM trichostatin A (TSA), or 25 µM 5′aza-cytidine for 36 h, and luciferase assays performed as described above. To block virus replication, 0.3 mg/ml PAA was added to the media upon infection.

### Quantitative PCR

The vector DNA in uninfected and infected cells was determined by quantitative real-time PCR. Cells (200,000) were seeded into each well of a 12-well plate and infected with MCMV-wt at an MOI of 0.5 or mock-treated. Total genomic DNA was extracted from uninfected and infected cells 36 h post infection (p.i.) using the DNeasy blood and tissue kit (Qiagen) according to the manufacturer's instructions. To quantify the ratio of pEpibo-luc-ori per cell (before and after infection), two PCRs were performed. One was specific for the pEpibo constructs, amplifying the blasticidin resistance gene (*bsr*) using the primers bsr-for-taqman 5′-CCTCATTGAA AGAGCAACGGCTAC-3′ and bsr-rev-taqman 5′-GCACCACGAGTTCTGCACAAGGT-3′ and the specific probe for bsr (5′-FAM- CATCTCTGAAGACTACAGCGTCGCCA-TAMRA-3′). The second PCR was specific for the cellular lamin B receptor (LBR) gene and used the primers LBR-for (5′-GGAAGTTTGTTGAGGGTGAAGTGGT-3′) and LBR-rev (5′-CCAGTTCGGTGCCATCTTTGTATTT-3′) and a specific probe for LBR (5′-FAM-CTGAGCCACG ACAACAAATCCCAGCTCTAC-TAMRA-3′). Vector DNA copy number was calculated by comparing the amplification with standard curves generated using the plasmids pEpibo-luc-ori or p06IET-LBRfl. DNA amplification was performed using the TaqMan 1000 RXN PCR core reagent kit (50 cycles of 95°C for 15 s and 60°C for 1 min) (Applied Biosystems).

### Standard PCR

For the recombination assay, standard PCR was used to detect the m74 gene with the primers m74-for 5′-TCCGGACAACGTCTTTCCC-3′ and m74-rev 5′-CCTCAGTTCCACTTGCCAGC-3′, which amplify 316 bp inside the m74 gene. The primers M54-for 5′-ATCATCCGTTGCATCTCGTTG-3 and M54-rev 5′-CGCCATCTGTATCCGTCCAT-3′ were used to detect the M54 gene.

### Fluorescence and immunofluorescence microscopy

The immediate-early protein, ie1 (a marker of infection) was detected using an indirect immunofluorescence assay. Cells were fixed in 50% acetone-50% methanol and stained using the monoclonal antibody, Croma 101 (kindly provided by Stipan Jonjic, University of Rijeka, Croatia) followed by a Cy3-coupled goat anti-mouse antibody (Dianova). Fluorescence and bright-field imaging were observed on an Axiovert 200 M system (Zeiss).

### Fluorescence *in situ* hybridization (FISH)

Metaphase spreads of proliferating NIH3T3:gfpscp-ori cells were obtained after 2–3 h incubation with demecolcemid (0.1 µg/ml, Sigma Aldrich) and resuspension in 0.91% (w/v) tri-sodium citrate-dihydrate hypotonic solution. Cells were fixed in cold Carnoy's fixative (methanol∶acetic acid; 3∶1 v/v) and stored for 1 week before further processing. FISH was performed as previously described with minor modifications [76]. Briefly, three different probes were generated to detect the pEpibo-gfpscp-ori plasmid. The first probe, detecting the *bsr* gene, was PCR-labeled with biotin-ATP (NEB, USA) (using the primers bsr-for-FISH: 5′-ATGGCCAAGCCTTTGTCTCA-3′ and bsr-rev-FISH 5′-AGATCGAGAAGCACCTGTCG-3′) and the second probe, detecting the *gfpscp* gene, was PCR-labeled with dig-UTP (Roche) (using the primers P(SV40)-for-FISH: 5′-TACCGAGCTCTTACGCGTGC-3′ and pA(SV40)-rev-FISH 5′-TAAGATACATTGATGAGTTTGGA-3′). The third probe, detecting the oriLyt fragment, was generated by nick translation using DEAC-UTP (Perkin Elmer, USA). Immunolabeling of the probes was achieved by adding streptavidin-Cy3.5 (Rockland, USA) and an anti-dig-fluorescein antibody (Roche Diagnostics, Germany). DNA was counterstained with DAPI. Images of the FISH slides were taken using an Axiovert 200 microscope (Zeiss, Germany) at 60× magnification.

### Western Blotting

Western Blot analysis was performed as previously described [41] using rat anti-HA (3F10, Roche), mouse anti-IE1/3 (CHROMA 101, kindly provided by Stipan Jonjic), mouse anti-gp48 (CHROMA 221, kindly provided by Stipan Jonjic) and rabbit anti-actin primary antibodies (20–33, Sigma). Horseradish peroxidase-coupled anti-mouse, anti-rat, and anti-rabbit immunoglobulin-specific antibodies (Dianova) were used for immunodetection followed by the ECL-Plus (Amersham) system.

### Statistical analysis

Statistical analysis was performed using GraphPad Prism4. Two-way ANOVA was used to analyze differences unless otherwise indicated. Asterisks denote statistically significant differences (*P<0.05; **P<0.01; ***P<0.001).

### Accession numbers

M50: GenBank ADD10432.1

m74: GenBank ADD10446.1

m48.2: GenBank ADD10430.1

MCMV Smith Strain: GenBank NC_004065

## Supporting Information

Figure S1 **Construction of the MCMV replicon vector.** A) The cloning of the highly repetitive oriLyt region was facilitated via a ‘pick-up’- cloning strategy. By this way, long repetitive and difficult to amplify regions can be cloned using a simple procedure. To this end, a PCR fragment was generated containing a kanamycin resistance gene (kanR) and the conditional bacteriophage origin R6Kγ, flanked by sequence homologies to a region upstream of the oriLyt in the MCMV genome The PCR fragment was recombined into the MCMV-BAC pSM3fr-FRT using homologous recombination, thereby also destroying the transcription initiation sites of the neighboring and overlapping M57 and m58 genes. The genomic *NheI* site at 95767 and the introduction of an additional *NheI* restriction facilitates the fragment containing the oriLyt (blue) with the kan^R^ and oriR6K to be cut out and re-circularized. The resulting plasmid pO6kan-MCMV-oriLyt was selected for oriR6K maintenance by growth in *E. coli* PIR1 under kanamycin selection. The oriLyt was now flanked by two *Mlu*I sites, which can be used for further subcloning of the oriLyt into the vector pEpibo resulting in the plasmid pEpibo-luc-ori (B) The map of the pEpibo-luc-ori plasmid with S/MAR (scaffold/matrix attachment region), bsr (Blasticidine resistance gene), P (Promoter), pA (poly A). (C) Mechanism of replicon vector induction. In uninfected cells (left) the reporter gene firefly luciferase (*luc*) of a replicon vector, which is an episomal plasmid harboring the oriLyt of MCMV, is silenced. During infection with MCMV(right), the virus provides all factors to replicate the oriLyt-containing plasmid. This results in the replication and reactivation of the episomal vector concomitantly with a strong induction of the silent FL reporter gene expression (yellow).
(TIF)

Figure S2 **Trans-activation of herpesviral promoters in superinfection.** To assay the induction capacity of different promoters under infection, as well as their general expression strength, bioluminescence analysis of transfected reporter plasmids were performed. NIH3T3 cells were transfected with either pEpibo-luc (with P(SV40)), or the respective constructs with the immediate early promoter P(CMVie), the early promoter P(M143) as well as the two late promoters P(M53) and P(M94). Respective plasmids were generated by PCR amplification of the promoters P(M143), P(M53) and P(M94) ( in general ∼500 bp upstream of the start ATG) inserting the restriction sites *Kpn*I and *Hind*III, with which they were further subcloned into the pEpibo-luc vector, exchanging the P(SV40) promoter. Transfection was normalized by the co-transfection of pTK-RL (Promega) encoding the renilla luciferase. Cells were infected with MCMV at an MOI of 0.1 and 24 h (A) or 48 h p.i. (B) a bioluminescence assay was performed. While the minimal P(SV40) is not influenced by MCMV infection, all other promoters responded according to their typical expression profiling in the viral context. (p.i. = post infection, RLU = relative light units). Thus the P(SV40) promoter is well suited to analyze the effect of the oriLyt sequence without taking additional promoter induction into account.
(TIF)

Figure S3 **Controls for 5′Aza-cytidine and TSA.** (A) A cell line containing an integrated GFPSCP driven by a CMVie promoter, namely NIH3T3:SB(GFPSCP) was used as positive control for 5′ aza-cytidine function. While the fluorescence of the fusion protein was partially silenced, addition of 5′Aza could recover fluorescence dose dependently. (B) Removal of silencing in the luc-ori cl.1 clone could be dose dependently recovered with various concentrations of TSA or by infection with MCMV at an MOI of 1. Bioluminescence assays were performed 36 h p.i. or drug administration, respectively.
(TIF)

Figure S4 **Limited co-operativity of TSA and infection on FL induction.** (A) Expression of MCMV proteins in TSA treated cells. NIH3T3 were either untreated, treated with 330 nM TSA (added by infection), or treated with 165 nM TSA (added 2 h before infection, according to [26], infected with MCMV at an MOI of 0.5. At 4, 12, 24, 48 h p.i. cells were harvested and subjected to western blot analysis. Both TSA conditions had an slight enhancing effect on immediate early gene expression at 4 h p.i., major expression differences can be detected at late stages for the proteins M50 and gp48.( B) To determine the effect of TSA on virus replication MEF cells, 2 h pretreated with 330 nM TSA or not, were infected with MCMV at an MOI of 0.1. TSA was maintained in the medium throughout the assay. Supernatants were collected over time and analyzed by standard plaque assay. Treatment of the cells with TSA had a negative effect on virus production by around 100 fold on day 5. (C) Luc-ori cl.1 cells were treated with 0 nM, 83 nM, 165 nM or 330 nM Trichostatin A and infected with MCMV at an MOI of 1 (hatched bars) or left uninfected (plain bars). 36 h p.i. FL induction was measured via bioluminescence assay. TSA treatment increased expression from luc-ori cells in a dose dependent manner. Infection in TSA treated cells did not reach luciferase reactivation of untreated cells. With increasing concentration of TSA inhibition of MCMV could be observed, thus only little synergistic effects on FL induction could be obtained.
(TIF)

Figure S5 **pEpibo-luc-ori is amplified upon MCMV infection.** NIH3T3 or luc-ori cl.1 - 4 were infected with MCMV at an MOI of 1 (hatched bar) or left untreated (white bar, mock).Quantitative realtime PCR was performed 36 h p.i. to determine copy numbers of pEpibo-luc-ori vectors by a PCR specific for the *bsr* coding sequence compared to a cellular single copy gene lamin B receptor (*lbr*). (p.i., post infection; ***: p<0.001, ns: p>0.05, Two-Way-ANOVA, depicted is mean+SD)
(TIF)

Figure S6 **Non-invasive bioluminescence imaging of VIOLA mice.** A) Bioluminescence imaging of luciferase expression in living VIOLA mice after intravenous injection of 1×10^6^ PFU MCMV-wt (right mouse). Shown is one of three mice in the experiment. As positive control 129X1/SvJ mice (129) were infected with 1×10^5^ PFU MCMV-luc (left mouse) and to assess the background bioluminescence 129 mice were infected with 1×10^6^ PFU MCMV-wt (middle mouse). The pseudocolor overlay represents the intensity of light emission, and thus the level of FL expression. At various times after administration of MCMV, the mice were imaged using a bioluminescence imaging system (Xenogen) to detect FL expression. 10 min after intraperitoneal injection of 2.5 mg luciferin ventral, lateral and dorsal images were collected for 5 min with maximum sensitivity. The mice were maintained under isoflurane anesthesia at 37°C. Strong signals of the positive control could be detected in 129 mice. However no signal from VIOLA mice was measurable. B) In order to exclude that failure of bioluminescence induction is due to lacking infection in the transgenic mice, viral titers in organ homogenates has been determined. VIOLA mice (which were negative in the invasive bioluminescence assay) were infected with 1×10^6^ PFU/ml MCMV-wt and sacrificed at day 5. Organs were homogenized and virus titers determined with standard plaque assay.
(TIF)

Figure S7 **Southern Blot analysis of VIOLA lines.** Genomic DNA of 129X1/SvJ or VIOLA mouse tails was extracted with the Qiagen Blood and Tissue Kit and 10 µg digested with *Pst*I. As control the vector pEpibo-luc-ori was linearized with *Pst*I and loaded at different amounts (100 ng, 10 ng, 1 ng, 100 pg, 10 pg, 1 pg). A 1.2 kb probe specifically detecting the firefly luciferase gene was created with the PCR dig probe synthesis kit (Roche). Southern Blot hybridization was mainly performed as previously described [77]. Detection of hybridized probes was performed with the dig-luminescent detection kit. Asterisks mark specific bands probed with an anti-luc-dig probe, indicating an integration of the pEpibo-luc-ori constructs in the VIOLA lines. In case of an episomal persistence, only one band on the size of the linearized control bands should appear. Thus in the VIOLA lines the pEpibo-luc-ori plasmid is least for the majority of the copies integrated into the mouse chromosomes. Note, that the mouse lines A and B possess different integration patterns.
(TIF)
