# Supplementary figures and images for: Cytomegalovirus Replicon-Based Regulation of Gene Expression In Vitro and In Vivo
Source: PLoS Pathog. 2012 Jun 7;8(6):e1002728. doi: 10.1371/journal.ppat.1002728 (PMC3369935; doi:10.1371/journal.ppat.1002728)

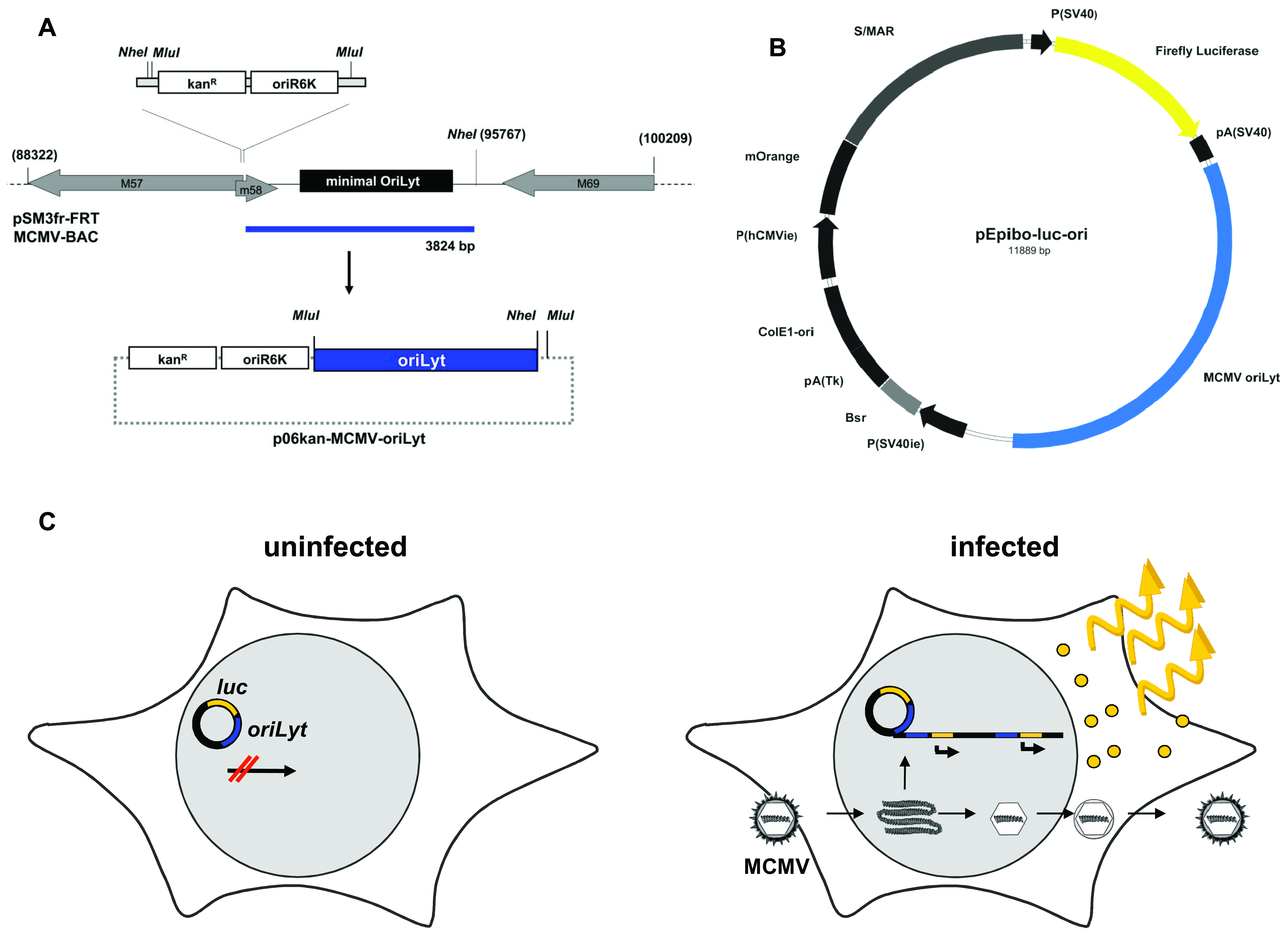

Supplement: Figure S1 — Construction of the MCMV replicon vector. A) The cloning of the highly repetitive oriLyt region was facilitated via a ‘pick-up’- cloning strategy. By this way, long repetitive and difficult to amplify regions can be cloned using a simple procedure. To this end, a PCR fragment was generated containing a kanamycin resistance gene (kanR) and the conditional bacteriophage origin R6Kγ, flanked by sequence homologies to a region upstream of the oriLyt in the MCMV genome The PCR fragment was recombined into the MCMV-BAC pSM3fr-FRT using homologous recombination, thereby also destroying the transcription initiation sites of the neighboring and overlapping M57 and m58 genes. The genomic NheI site at 95767 and the introduction of an additional NheI restriction facilitates the fragment containing the oriLyt (blue) with the kanR and oriR6K to be cut out and re-circularized. The resulting plasmid pO6kan-MCMV-oriLyt was selected for oriR6K maintenance by growth in E. coli PIR1 under kanamycin selection. The oriLyt was now flanked by two MluI sites, which can be used for further subcloning of the oriLyt into the vector pEpibo resulting in the plasmid pEpibo-luc-ori (B) The map of the pEpibo-luc-ori plasmid with S/MAR (scaffold/matrix attachment region), bsr (Blasticidine resistance gene), P (Promoter), pA (poly A). (C) Mechanism of replicon vector induction. In uninfected cells (left) the reporter gene firefly luciferase (luc) of a replicon vector, which is an episomal plasmid harboring the oriLyt of MCMV, is silenced. During infection with MCMV(right), the virus provides all factors to replicate the oriLyt-containing plasmid. This results in the replication and reactivation of the episomal vector concomitantly with a strong induction of the silent FL reporter gene expression (yellow). (TIF) [file ppat.1002728.s001.tif]

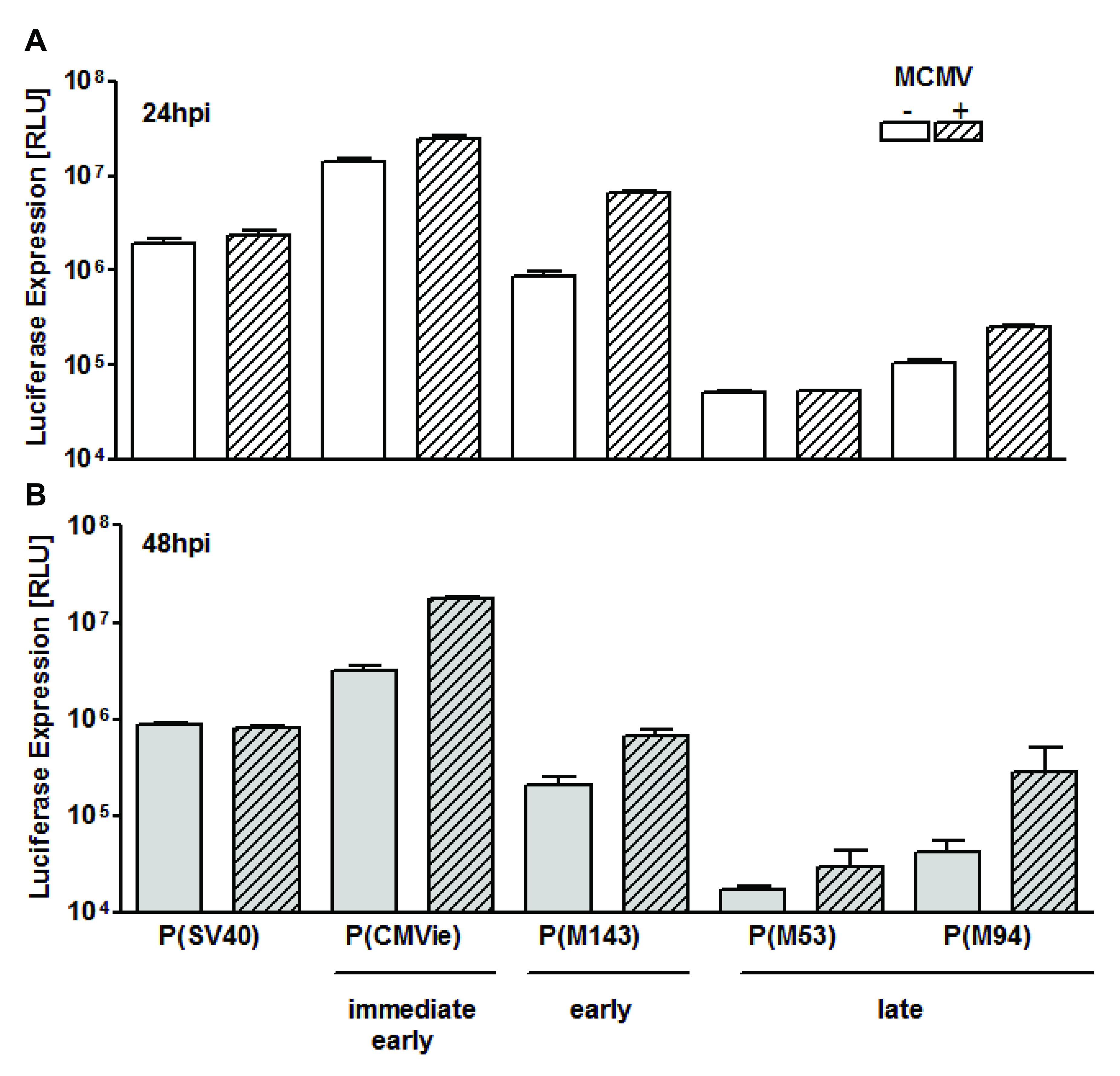

Supplement: Figure S2 — Trans-activation of herpesviral promoters in superinfection. To assay the induction capacity of different promoters under infection, as well as their general expression strength, bioluminescence analysis of transfected reporter plasmids were performed. NIH3T3 cells were transfected with either pEpibo-luc (with P(SV40)), or the respective constructs with the immediate early promoter P(CMVie), the early promoter P(M143) as well as the two late promoters P(M53) and P(M94). Respective plasmids were generated by PCR amplification of the promoters P(M143), P(M53) and P(M94) ( in general ∼500 bp upstream of the start ATG) inserting the restriction sites KpnI and HindIII, with which they were further subcloned into the pEpibo-luc vector, exchanging the P(SV40) promoter. Transfection was normalized by the co-transfection of pTK-RL (Promega) encoding the renilla luciferase. Cells were infected with MCMV at an MOI of 0.1 and 24 h (A) or 48 h p.i. (B) a bioluminescence assay was performed. While the minimal P(SV40) is not influenced by MCMV infection, all other promoters responded according to their typical expression profiling in the viral context. (p.i. = post infection, RLU = relative light units). Thus the P(SV40) promoter is well suited to analyze the effect of the oriLyt sequence without taking additional promoter induction into account. (TIF) [file ppat.1002728.s002.tif]

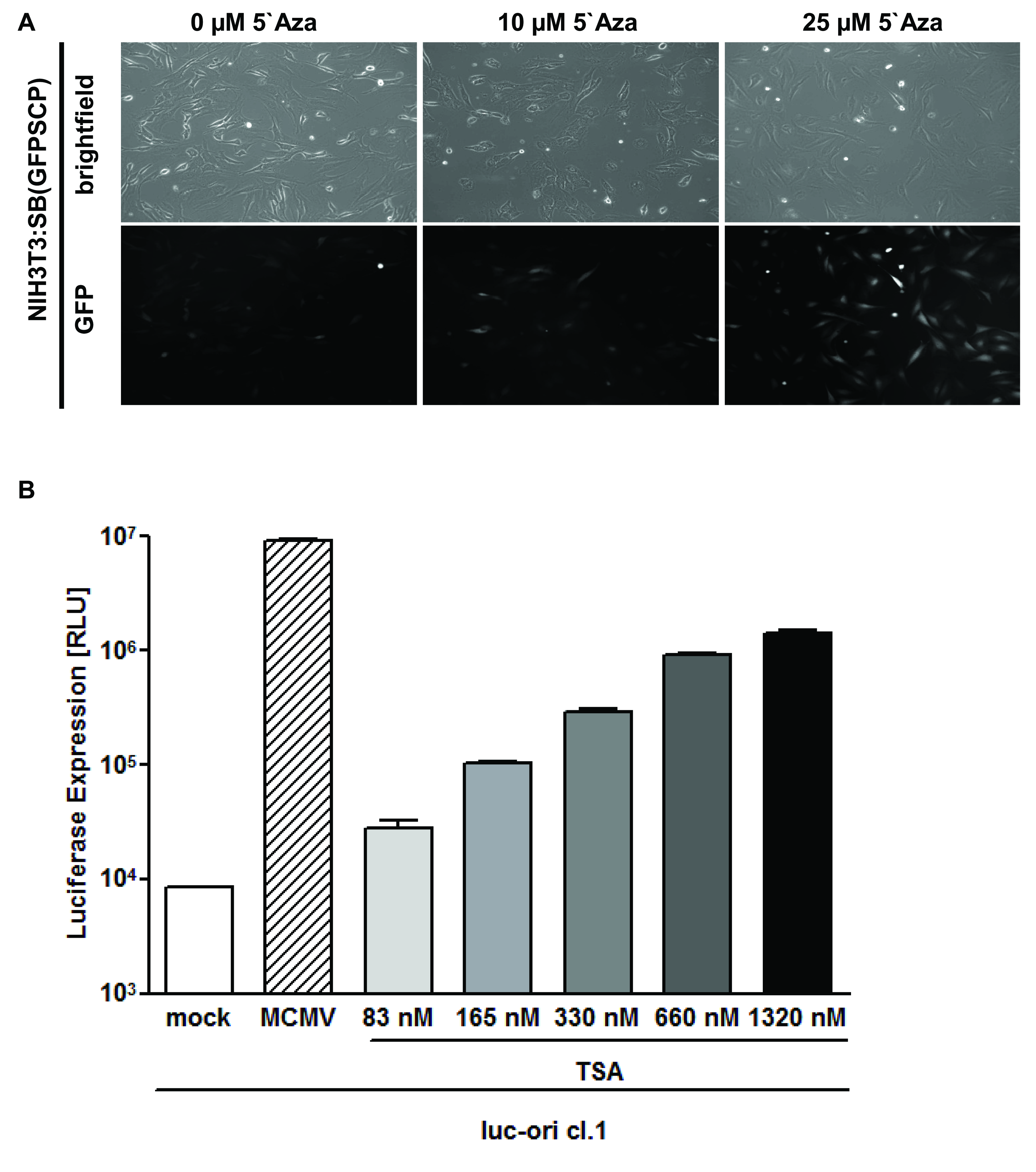

Supplement: Figure S3 — Controls for 5′Aza-cytidine and TSA. (A) A cell line containing an integrated GFPSCP driven by a CMVie promoter, namely NIH3T3:SB(GFPSCP) was used as positive control for 5′ aza-cytidine function. While the fluorescence of the fusion protein was partially silenced, addition of 5′Aza could recover fluorescence dose dependently. (B) Removal of silencing in the luc-ori cl.1 clone could be dose dependently recovered with various concentrations of TSA or by infection with MCMV at an MOI of 1. Bioluminescence assays were performed 36 h p.i. or drug administration, respectively. (TIF) [file ppat.1002728.s003.tif]

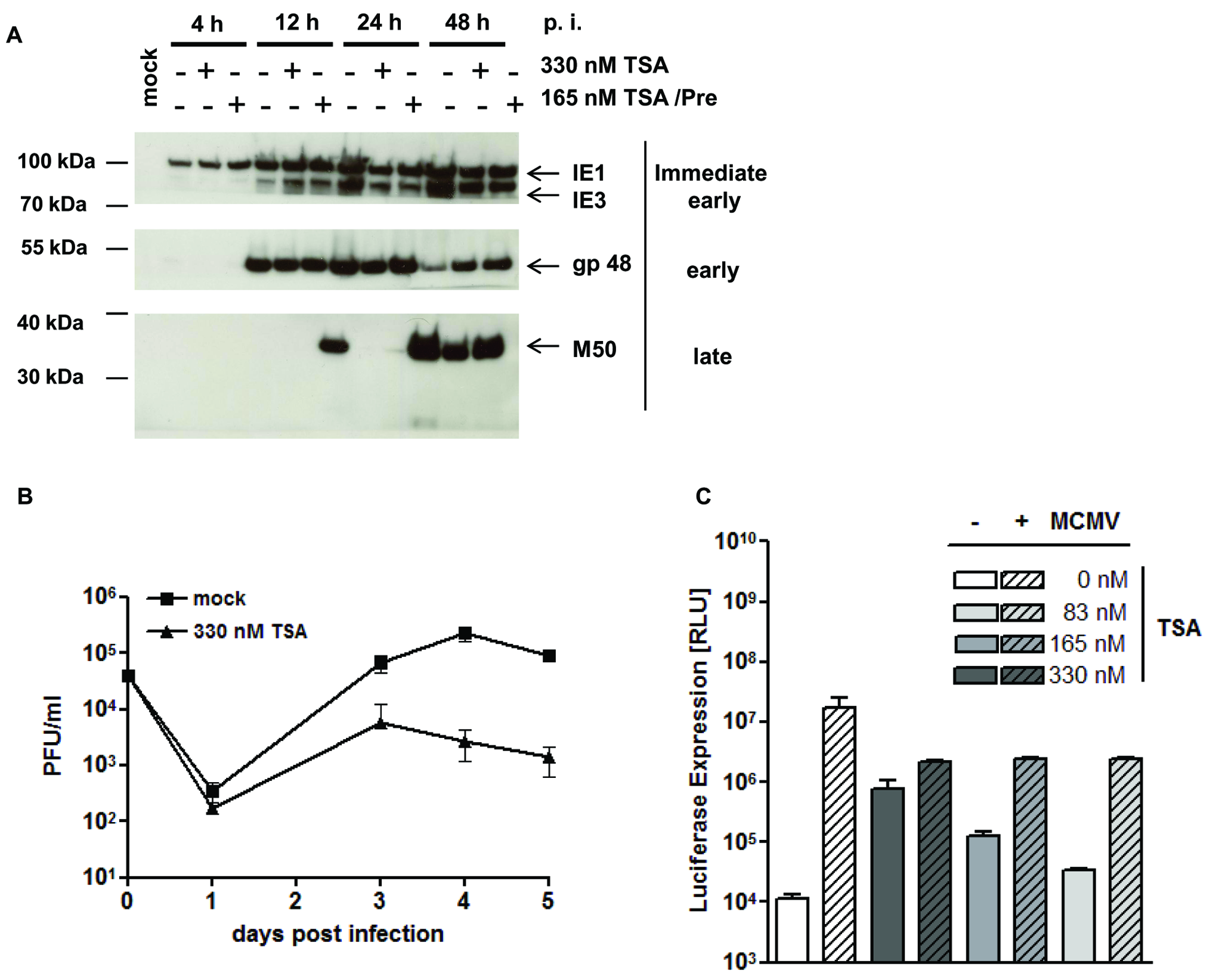

Supplement: Figure S4 — Limited co-operativity of TSA and infection on FL induction. (A) Expression of MCMV proteins in TSA treated cells. NIH3T3 were either untreated, treated with 330 nM TSA (added by infection), or treated with 165 nM TSA (added 2 h before infection, according to [26], infected with MCMV at an MOI of 0.5. At 4, 12, 24, 48 h p.i. cells were harvested and subjected to western blot analysis. Both TSA conditions had an slight enhancing effect on immediate early gene expression at 4 h p.i., major expression differences can be detected at late stages for the proteins M50 and gp48.( B) To determine the effect of TSA on virus replication MEF cells, 2 h pretreated with 330 nM TSA or not, were infected with MCMV at an MOI of 0.1. TSA was maintained in the medium throughout the assay. Supernatants were collected over time and analyzed by standard plaque assay. Treatment of the cells with TSA had a negative effect on virus production by around 100 fold on day 5. (C) Luc-ori cl.1 cells were treated with 0 nM, 83 nM, 165 nM or 330 nM Trichostatin A and infected with MCMV at an MOI of 1 (hatched bars) or left uninfected (plain bars). 36 h p.i. FL induction was measured via bioluminescence assay. TSA treatment increased expression from luc-ori cells in a dose dependent manner. Infection in TSA treated cells did not reach luciferase reactivation of untreated cells. With increasing concentration of TSA inhibition of MCMV could be observed, thus only little synergistic effects on FL induction could be obtained. (TIF) [file ppat.1002728.s004.tif]

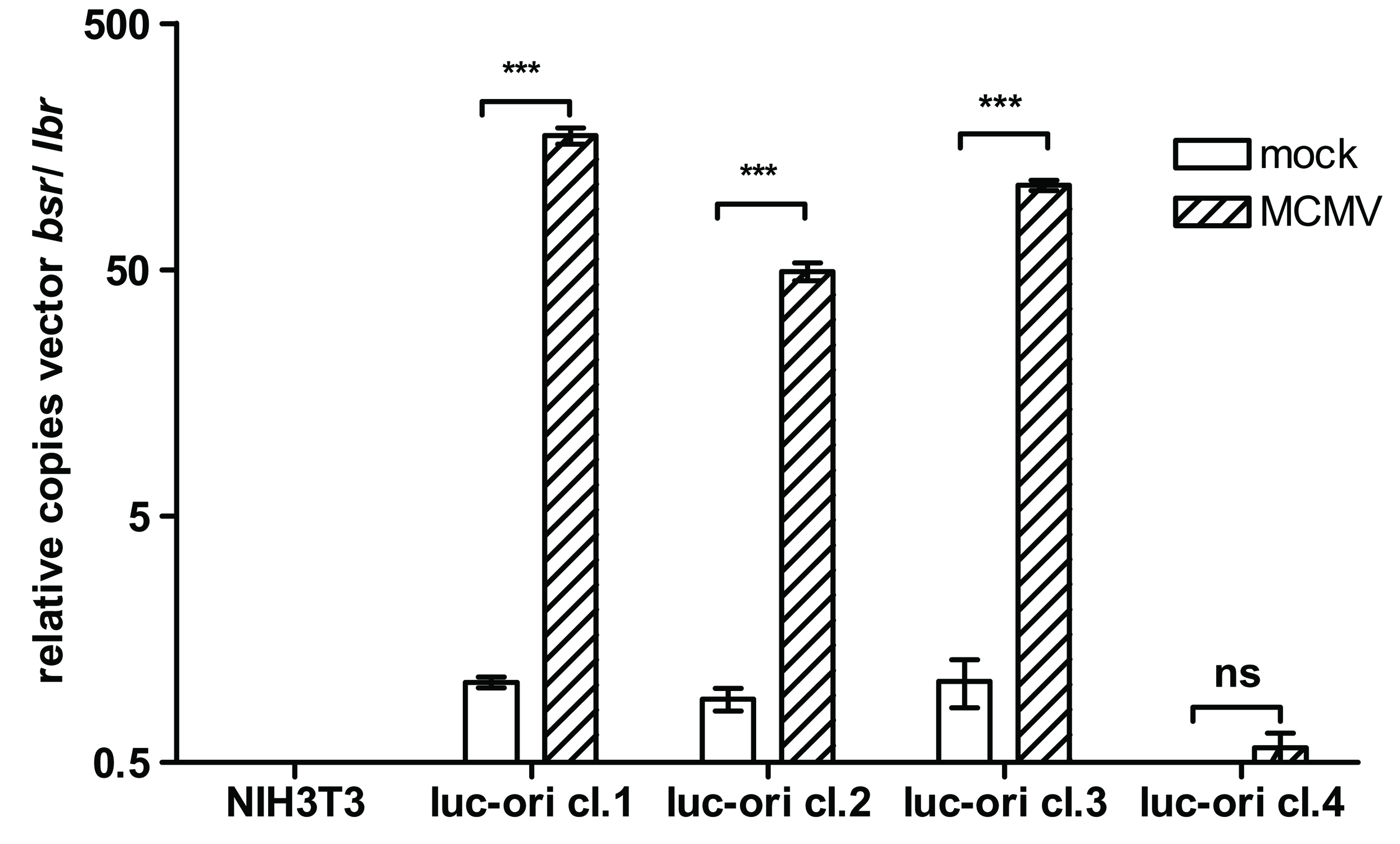

Supplement: Figure S5 — pEpibo-luc-ori is amplified upon MCMV infection. NIH3T3 or luc-ori cl.1 - 4 were infected with MCMV at an MOI of 1 (hatched bar) or left untreated (white bar, mock).Quantitative realtime PCR was performed 36 h p.i. to determine copy numbers of pEpibo-luc-ori vectors by a PCR specific for the bsr coding sequence compared to a cellular single copy gene lamin B receptor (lbr). (p.i., post infection; ***: p<0.001, ns: p>0.05, Two-Way-ANOVA, depicted is mean+SD) (TIF) [file ppat.1002728.s005.tif]

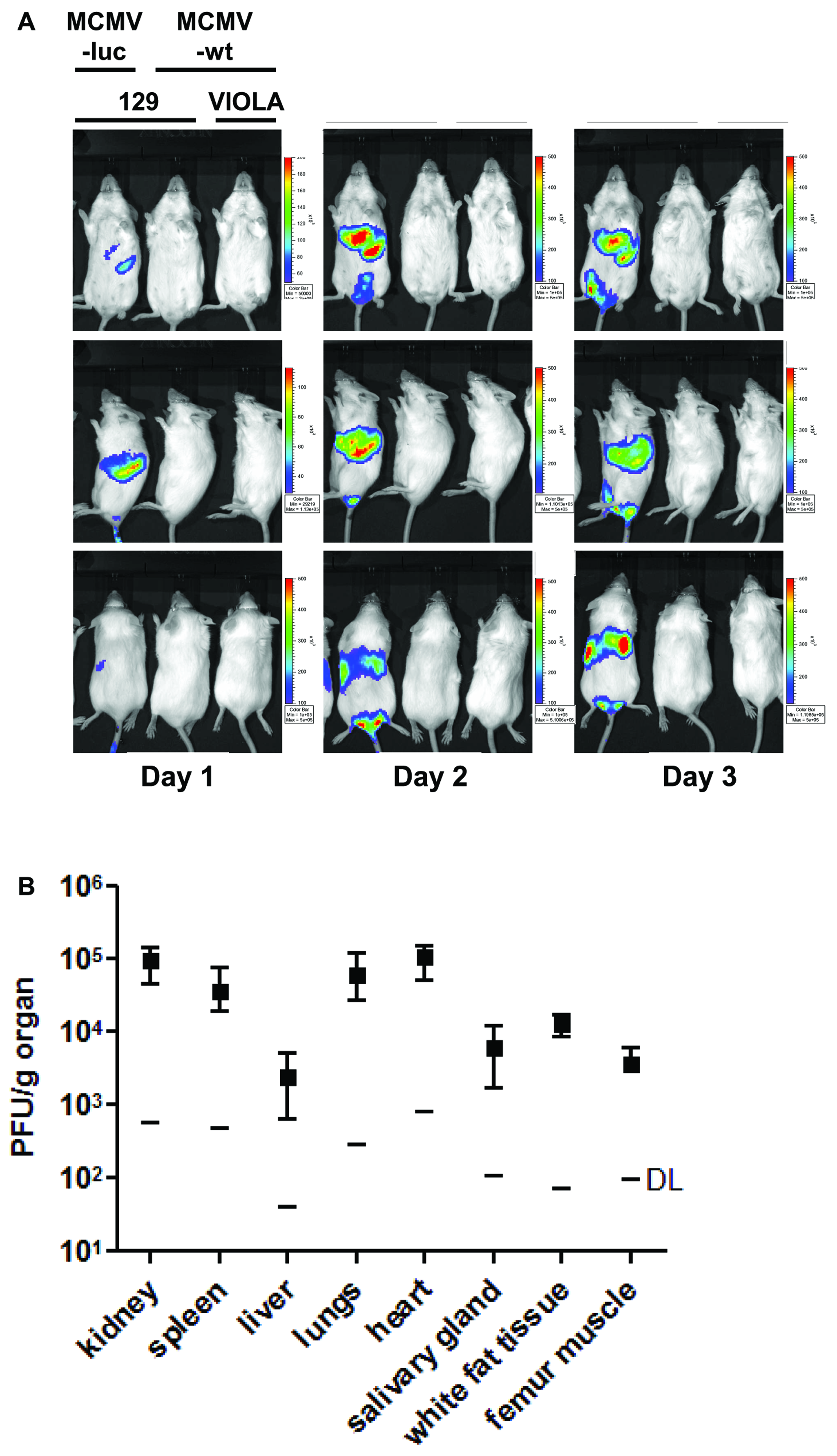

Supplement: Figure S6 — Non-invasive bioluminescence imaging of VIOLA mice. A) Bioluminescence imaging of luciferase expression in living VIOLA mice after intravenous injection of 1×10^6^ PFU MCMV-wt (right mouse). Shown is one of three mice in the experiment. As positive control 129X1/SvJ mice (129) were infected with 1×10^5^ PFU MCMV-luc (left mouse) and to assess the background bioluminescence 129 mice were infected with 1×10^6^ PFU MCMV-wt (middle mouse). The pseudocolor overlay represents the intensity of light emission, and thus the level of FL expression. At various times after administration of MCMV, the mice were imaged using a bioluminescence imaging system (Xenogen) to detect FL expression. 10 min after intraperitoneal injection of 2.5 mg luciferin ventral, lateral and dorsal images were collected for 5 min with maximum sensitivity. The mice were maintained under isoflurane anesthesia at 37°C. Strong signals of the positive control could be detected in 129 mice. However no signal from VIOLA mice was measurable. B) In order to exclude that failure of bioluminescence induction is due to lacking infection in the transgenic mice, viral titers in organ homogenates has been determined. VIOLA mice (which were negative in the invasive bioluminescence assay) were infected with 1×10^6^ PFU/ml MCMV-wt and sacrificed at day 5. Organs were homogenized and virus titers determined with standard plaque assay. (TIF) [file ppat.1002728.s006.tif]

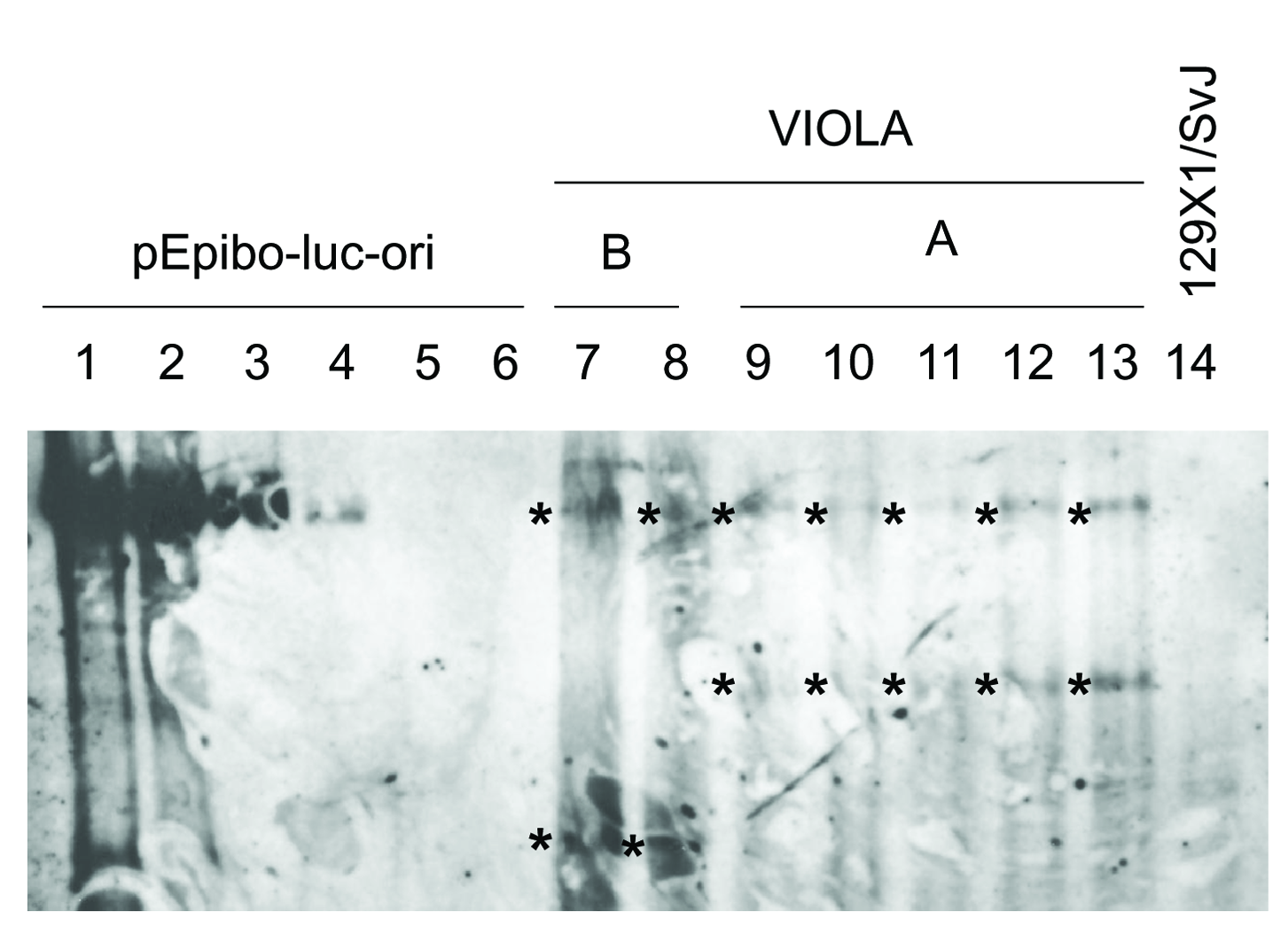

Supplement: Figure S7 — Southern Blot analysis of VIOLA lines. Genomic DNA of 129X1/SvJ or VIOLA mouse tails was extracted with the Qiagen Blood and Tissue Kit and 10 µg digested with PstI. As control the vector pEpibo-luc-ori was linearized with PstI and loaded at different amounts (100 ng, 10 ng, 1 ng, 100 pg, 10 pg, 1 pg). A 1.2 kb probe specifically detecting the firefly luciferase gene was created with the PCR dig probe synthesis kit (Roche). Southern Blot hybridization was mainly performed as previously described [77]. Detection of hybridized probes was performed with the dig-luminescent detection kit. Asterisks mark specific bands probed with an anti-luc-dig probe, indicating an integration of the pEpibo-luc-ori constructs in the VIOLA lines. In case of an episomal persistence, only one band on the size of the linearized control bands should appear. Thus in the VIOLA lines the pEpibo-luc-ori plasmid is least for the majority of the copies integrated into the mouse chromosomes. Note, that the mouse lines A and B possess different integration patterns. (TIF) [file ppat.1002728.s007.tif]
